# Use of a Sibling Subtraction Method for Identifying Causal Mutations in *Caenorhabditis elegans* by Whole-Genome Sequencing

**DOI:** 10.1534/g3.117.300135

**Published:** 2017-12-12

**Authors:** Braveen B. Joseph, Nicolas A. Blouin, David S. Fay

**Affiliations:** * Idea Networks for Biomedical Research Excellence (INBRE) Bioinformatics Core, University of Wyoming, Laramie, Wyoming 82071; † Department of Molecular Biology, College of Agriculture and Natural Resources, University of Wyoming, Laramie, Wyoming 82071

**Keywords:** *C. elegans*, counterselection, NimA kinases, *peel-1*, suppressor screens whole-genome sequencing

## Abstract

Whole-genome sequencing (WGS) is an indispensable tool for identifying causal mutations obtained from genetic screens. To reduce the number of causal mutation candidates typically uncovered by WGS, *Caenorhabditis elegans* researchers have developed several strategies. One involves crossing N2-background mutants to the polymorphic Hawaiian (HA) strain, which can be used to simultaneously identify mutant strain variants and obtain high-density mapping information. This approach, however, is not well suited for uncovering mutations in complex genetic backgrounds, and HA polymorphisms can alter phenotypes. Other approaches make use of DNA variants present in the initial background or introduced by mutagenesis. This information is used to implicate genomic regions with high densities of DNA lesions that persist after backcrossing, but these methods can provide lower resolution than HA mapping. To identify suppressor mutations using WGS, we developed an approach termed the sibling subtraction method (SSM). This method works by eliminating variants present in both mutants and their nonmutant siblings, thus greatly reducing the number of candidates. We used this method with two members of the *C. elegans* NimA-related kinase family, *nekl-2* and *nekl-3*. Combining weak aphenotypic alleles of *nekl-2* and *nekl-3* leads to penetrant molting defects and larval arrest. We isolated ∼50 suppressors of *nekl-2*; *nekl-3* synthetic lethality using F1 clonal screening methods and a *peel-1*–based counterselection strategy. When applied to five of the suppressors, SSM led to only one to four suppressor candidates per strain. Thus SSM is a powerful approach for identifying causal mutations in any genetic background and provides an alternative to current methods.

Whole-genome sequencing (WGS) was first used to identify causal mutations in *Caenorhabditis* *elegans* nearly 10 yr ago (Sarin *et al.* 2008), and this approach has been progressively refined and applied to a growing number of organisms (Schneeberger and Weigel 2011; Leshchiner *et al.* 2012; Obholzer *et al.* 2012; James *et al.* 2013; Moresco *et al.* 2013; Hu 2014; Schneeberger 2014; Doitsidou *et al.* 2016). One complication of WGS is that many hundreds or thousands of variants are typically detected in individual mutant strains, thus making it difficult to pinpoint the causal mutation that is responsible for the observed phenotype. Because the large majority of mutations that alter phenotypes lead to changes in the primary sequence of proteins (Sarin *et al.* 2008), filtering steps can be applied so that only DNA variants that alter coding sequences are considered. Nevertheless, in the absence of other information, this can still result in large numbers of exonic variants that must be experimentally tested.

Several WGS strategies have been developed to reduce the number of candidate variants by simultaneously providing mapping data on the causal mutation, an approach termed “mapping by sequencing” (Schneeberger and Weigel 2011; Zuryn and Jarriault 2013; Schneeberger 2014; Doitsidou *et al.* 2016). In *C. elegans*, a widely used approach makes use of the Hawaiian (HA) variant, CB4856, which differs from the field-standard N2 Bristol strain by >100,000 polymorphisms (C. elegans Sequencing Consortium 1998; Wicks *et al.* 2001; Hillier *et al.* 2008; Doitsidou *et al.* 2010; Vergara *et al.* 2014). Typically, mutants generated in the N2 background are crossed to the HA strain to generate N2/HA hybrids, which are then allowed to self-fertilize. Homozygous mutant progeny of N2/HA hermaphrodites are then isolated and subjected to WGS and variant identification. N2/HA polymorphisms can then be exploited to map mutations to genomic regions that are homozygous for N2-specific variants, thereby greatly reducing the number of causal variants to be considered (Doitsidou *et al.* 2010). Variations on HA mapping have also been developed to facilitate the mapping and identification of diverse allele types (Smith *et al.* 2016). This approach, however, has two major caveats. One is that it is not easily applied to complex genotypes, such as mutations that alter phenotypes only when one or more additional mutant loci are present in the background. The other is that genetic differences between N2 and HA can lead to changes in the expression of phenotypes in ways that are not predictable (Doroszuk *et al.* 2009; Reddy *et al.* 2009; Bendesky *et al.* 2012; Rodriguez *et al.* 2012; Green *et al.* 2013; Pollard and Rockman 2013; Glater *et al.* 2014; Snoek *et al.* 2014; Doitsidou *et al.* 2016; Kamkina *et al.* 2016; Singh *et al.* 2016).

As an alternative, the method known as ethyl methanesulfonate (EMS) density mapping does not require use of the HA strain and relies on the ability to detect signature changes to DNA that are induced by the commonly used mutagen EMS (Drake and Baltz 1976; Flibotte *et al.* 2010; Sarin *et al.* 2010). After serial backcrossing, strains are subjected to WGS, and genomic regions that contain higher densities of EMS lesions (such as those flanking causal mutations) are identified (Zuryn *et al.* 2010). Because the density of EMS-induced lesions is relatively low compared with the number of polymorphisms between N2 and HA, this method may yield substantially lower mapping resolution than the HA method (Doitsidou *et al.* 2016).

Another approach that circumvents use of the HA strain, variant discovery mapping (VDM), also relies on variant frequencies to identify chromosomal regions of interest, but uses both EMS and non-EMS variants (Minevich *et al.* 2012; Cheesman *et al.* 2016). Thus, VDM may improve mapping resolution relative to that of EMS density mapping, but may provide less information than HA mapping methods (Doitsidou *et al.* 2016).

Here, we describe the sibling subtraction method (SSM), which is an alternative approach for WGS analysis that does not require use of the HA strain and does not conceptually rely on variant mapping methods. We applied this strategy to identify suppressors of synthetically lethal *nekl-2*; *nekl-3* double mutants, which arrest as larvae because of defects in molting (Yochem *et al.* 2015; Lazetic and Fay 2017). Our results indicate that this method can be used to reduce the number of candidate causal variants to as few as one or two coding change candidates in most cases, thus providing a powerful alternative to current approaches. This study also highlights the utility of using synthetically lethal combinations of weak aphenotypic alleles as a genetic background for suppressor screening, and includes a description of a counterselection approach to increase the efficiency of genetic suppressor screens.

## Materials and Methods

### Strains and maintenance

*C. elegans* strains were maintained according to standard protocols (Stiernagle 2006) and were propagated at 22° unless otherwise stated. Strains used in this study include N2 Bristol (wild type), WY1145 [*nekl-2(fd81)*; *nekl-3(gk894345)*; *fdEx286* (pDF153, *nekl-3(+)*; pTG96, SUR-5::GFP)] (Lazetic and Fay 2017), WY1208 [*nekl-2(fd81)*; *nekl-3(gk894345)*; *fd130*], WY1209 [*nekl-2(fd81)*; *fd131*; *nekl-3(gk894345)*], WY1210 [*nekl-2(fd81)*; *fd132*; *nekl-3(gk894345)*], WY1211 [*nekl-2(fd81)*; *fd133*; *nekl-3(gk894345)*], WY1217 [*nekl-2(fd81)*; *nekl-3(gk894345)*; *fd139*], WY1232 [*nekl-2(fd81)*; *nekl-3(gk894345)*; *fdEx286*; *fdEx297* (pTG96.2, SUR-5::RFP)], and WY1255 [*nekl-2(fd81)*; *nekl-3*; *fdEx303* (pDF153, *nekl-3(+)*; pTG96, SUR-5::GFP, PMA122, P*_hsp16.41_:peel-1*)] (Seidel *et al.* 2011). For additional strains, see Supplemental Material, Table S1.

### Suppressor screens

Suppressors were obtained through an F1 clonal screen following standard EMS mutagenesis (Kutscher and Shaham 2014) of strain WY1145. Suppressed worms were detected by their ability to propagate in the absence of the GFP-marked *nekl-3*–rescuing array, *fdEx286*, and further confirmed by backcrossing to WY1145. For the *peel-1* counterselection screen, strain WY1255 was mutagenized with EMS and individual P0s were placed on large NGM plates, grown for two generations, and then heat shocked to eliminate array-containing F2s. After 4 d, plates were heat shocked to eliminated rare escapers and plates were then screened 3–4 d later for the presence of propagating (GFP^−^) suppressed worms.

### Genetic analysis of suppressors

For each backcross, suppressed hermaphrodites were crossed to WY1145 males, GFP^+^ F1 cross-progeny hermaphrodites were individually cloned, and GFP^−^ F2 suppressed animals were isolated. From these crosses, the frequencies of GFP^−^ males were scored in the F1 generation to determine if the suppressors were either dominant or on LGX. To distinguish between dominant mutations and recessive mutations on LGX, we crossed suppressors to WY1232 (GFP^+^ RFP^+^) males and scored for the presence of RFP^+^ hermaphrodites in the F1 generation. To determine if the dominant mutation *fd132* (WY1210) was on LGX, WY1210 hermaphrodites were crossed to WY1232 males, and viable RFP^+^ F1 males were then crossed to WY1145 hermaphrodites. We then scored for the presence of suppressed F1 males (GFP^−^ RFP^−^ or GFP^−^ RFP^+^). Although we observed >100 GFP^+^ RFP^−^ and/or GFP^+^ RFP^+^ males resulting from this cross, we failed to observe any GFP^−^ RFP^+^ or GFP^−^RFP^−^ males, indicating linkage to LGX. Additionally, we picked F1 GFP^+^ RFP^+^ hermaphrodites singly to plates and scored their F2 progeny. We observed 100% (*n* = 40) of GFP^+^ RFP^+^ hermaphrodites to segregate suppressed progeny, further confirming the location of *fd132* on LGX.

### Preparation of DNA

After either two (WY1208) or four (WY1209, WY1210, WY1211, and WY1217) backcrosses, suppressed strains were mated to WY1145 males and several F1 heterozygous (GFP^+^) cross-progeny were allowed to self-fertilize. Then, 50–100 F2 self-progeny were subsequently cloned to individual plates to obtain the *sup/sup* (*Sup*), *sup/+*, and *+/+* (*Non-Sup*) genotypes. Thus, sequencing analysis was carried on strains that were backcrossed a total of 3× (WY1208) or 5× (WY1209, WY1210, WY1211, and WY1217). DNA was prepared as previously described using the Gentra Puregene Tissue Kit (Doitsidou *et al.* 2010). The following numbers of independent isolates were used to generate the DNA pools: WY1208 (*Sup*, 10; *Non-Sup*, 15), WY1209 (*Sup*, 10; *Non-Sup*, 10), WY1210 (*Sup*, 10; *Non-Sup*, 13), WY1211 (*Sup*, 10; *Non-Sup*, 5), and WY1217 (*Sup*, 5; *Non-Sup*, 8).

### Variant detection

Paired-end libraries were prepared for each strain and sequenced on an Illumina HiSeq 2000. Resulting reads were analyzed via SSM as described in the *Results*. Briefly, our workflow was adapted from CloudMap and incorporated tools including Trimmomatic, BWA, SAMtools, Genome Analysis Toolkit (GATK), Generate pileup, VarScan, SnpEff, SnpSift, and Integrative Genomics Viewer implemented on the UseGalaxy.org platform (Li and Durbin 2009; Li *et al.* 2009; McKenna *et al.* 2010; Cingolani *et al.* 2012a,b; Koboldt *et al.* 2012; Bolger *et al.* 2014; Afgan *et al.* 2016). A detailed description of variant analysis is provided in the Supplemental Methods in File S1.

### Data Availability

Reads for all strains used in this study are deposited in NCBI's Sequence Read Archive under BioProject ID PRJNA415825. Code (workflows) used to generate the results in our SSM method are available as *SSM Variant Detection* and *SSM Variant Subtraction* and can be accessed through the shared workflows at usegalaxy.org/workflows (owner: bjoseph). Specific software versions and parameters for workflows are provided in Supplementary Methods in File S1.

### RNAi

dsRNAs corresponding to exons in candidate suppressor genes were prepared using standard PCR methods followed by T7 RNA synthesis (for primer sequences see Supplemental Methods, File S1) and were injected at 0.8–1.0 µg/µl into strain WY1145 (Ahringer 2005). GFP^−^ F1 progeny were scored, together with noninjected controls, for each experiment. For WY1217, injection of dsRNA corresponding to exon 11 of B0302.1 (1033 bp fragment) led to 18.6% (*n* = 1112) adult viability *vs.* 0.7% (*n* = 279) in noninjected controls (*P* < 0.0001). For WY1209, injection of dsRNA corresponding to exons 9–11 of F56D12.6a (970 bp fragment) led to 16.5% (*n* = 1054) adult viability *vs.* 1.9% (*n* = 210) in noninjected controls (*P* < 0.0001).

### CRISPR/Cas9

CRISPR/Cas9 RNPs were injected into strain WY1145 using *dpy-10* co-CRISPR methods (Arribere *et al.* 2014; Paix *et al.* 2014, 2015), and Rol and Dpy progeny were monitored for suppression over two generations (for crRNA and other oligonucleotide sequences see File S1). In cases where mutations in the candidate suppressors led to the premature termination of transcripts (C04A11.4, F56D12.6, and B0302.1), we generated breaks within 45 bp of the candidate lesion and allowed nonhomologous repair mechanisms to generate new stop codons or frameshifts. This led to the isolation of three new alleles of C04A11.4 (*fd208*, 1 bp deletion; *fd209*, 2 bp deletion; *fd210*, 8 bp deletion), two new alleles of F56D12.6 (*fd211*, 13 bp deletion; *fd212*, 10 bp deletion), and three new alleles of B0302.1 (*fd213*, 16 bp insertion; *fd214*, 7 bp deletion; *fd215*, multiple substitutions and insertions). For specific sequences, see Table S2. For the essential gene F48E8.5, we used a repair template to introduce the specific lesion identified by WGS. This led to the isolation of two independent alleles (*fd216* and *fd217*), both of which contained the identical (G→A) nucleotide substitution and also displayed suppression.

### Transgenic rescue

Fosmids containing a wild-type copy of the candidate gene were injected with SUR-5::GFP into *nekl-2*; *nekl-3*; *sup* adults, and GFP^+^ F1 progeny were scored for molting defects. In some cases, incomplete rescue (desuppression) led to our ability to score progeny from the F2 generation, although we were unable to obtain stably transmitting lines. For strain WY1208, we injected fosmids corresponding to C04A11.4 (WRM0620dD12, WRM0632aG02, and WRM0610cA04, 2–6 ng/µl each + SUR-5::GFP [pTG96], 100 ng/µl). Suppression by *fd130* in WY1208 led to 83% viability (17% arrest; Table 1). Following injection of WY1208, 80% of GFP^+^ F1 larvae (*n* = 25; *P* < 0.0001) arrested with molting defects, and a similar trend was observed in the F2 progeny from several viable transmitting F1s (83% arrest, *n* = 37; *P* < 0.0001). In contrast, injection of this fosmid mix into wild type showed no deleterious effects on larval development or adult viability. WY1217 was injected with fosmids corresponding to candidate B0302.1 (WRM061cD03 and WRM0612dE01, 6 ng/µl each + SUR-5::GFP [pTG96], 100 ng/µl). Whereas the *fd139* mutation led to 76% viability in this strain (24% arrest), we observed arrest in 93% of GFP^+^ F1s (*n* = 30; *P* < 0.0001). For strain WY1211 (*fd133*) we injected a mix of fosmids corresponding to F48E8.5 and an RFP marker (WRM0618bG08, WRM064bA06, and WRM0618aG08, ∼6 ng/µl each + SUR-5::RFP [pTG96.2], 100 ng/µl) into WY1211 worms that also carried the *fdEx286 nekl-3*^+^ GFP^+^ rescuing array. Three stably transmitting GFP^+^ RFP^+^ lines were then for scored viability in the F3 generation. Arrest among the GFP^−^ RFP^+^ animals occurred at frequencies of 92% (*n* = 210), 95% (*n* = 128), and 97% (*n* = 181) for the three lines tested, respectively, whereas arrest among GFP^−^ RFP^−^ worms was 63% (*n* = 223; *P* < 0.0001). Furthermore, <10% of GFP^+^ RFP^+^ progeny underwent arrest (*n* > 200 for each strain), indicating that the arrays were not highly toxic. *P* values were determined using the N-1 chi-squared test.

**Table 1:** Strains used for sequencing analysis

Strain Name	Allele	% Suppression (*n*)	Dominant/Recessive	Autosome/LGX
WY1208	*fd130*	83 (223)	Recessive	LGX
WY1209	*fd131*	87 (360)	Recessive	Autosome
WY1210	*fd132*	50 (276)	Dominant	LGX
WY1211	*fd133*	35 (422)	Recessive	Autosome
WY1217	*fd139*	76 (235)	Recessive	LGX

For details on genetic analyses see *Materials and Methods*.

## Results

### An F1 clonal screen to identify suppressors of nekl-2; nekl-3 synthetic lethality

**Figure 1:**
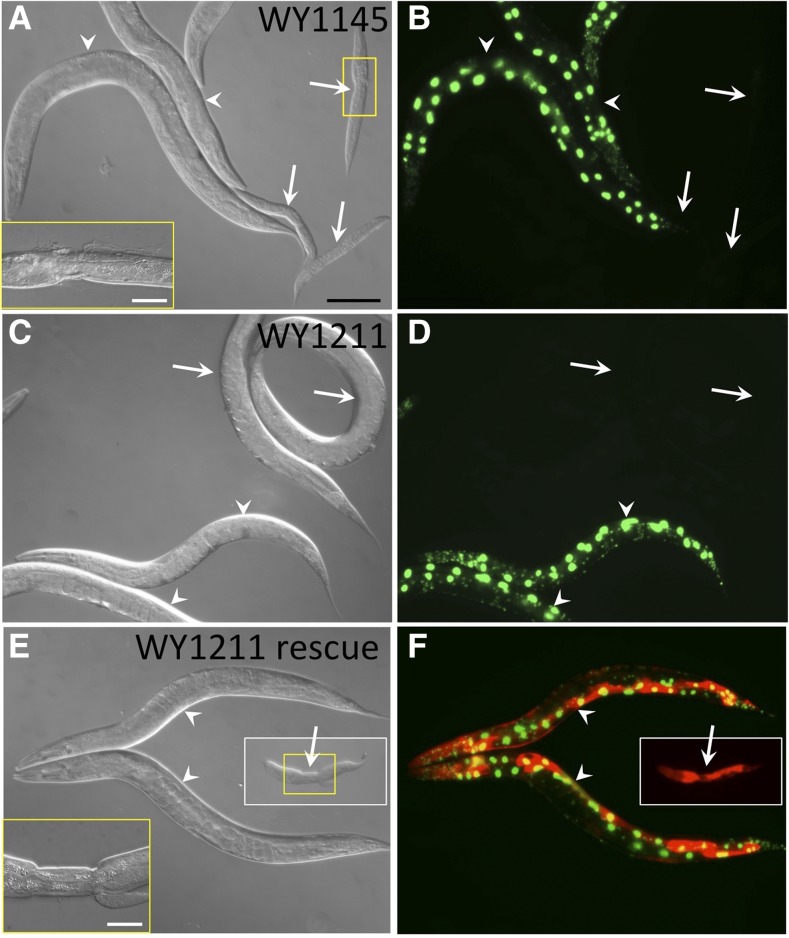
Phenotypes of molting-defective and suppressed strains. (A, C, and E) DIC and (B, D, and F) fluorescence images of strains WY1145 [*nekl-2(fd81)*; *nekl-3(gk894345)*] and WY1211 [*nekl-2(fd81)*; *paa-1(fd134)*; *nekl-3(gk894345)*]. GFP^+^ worms carry an extrachromosomal array (*fdEx286*) that expresses wild-type *nekl-3* and SUR-5::GFP. (A and B) Whereas GFP^−^ worms (white arrows) in the starting strain, WY1145, arrest uniformly with molting defects. (C and D) GFP^−^ worms in the suppressed strain, WY1211, can reach adulthood. (E and F) Suppression is reverted in strain WY1211 by the expression of wild-type *paa-1* from a fosmid, which is carried by an independent extrachromosomal array marked with SUR-5::RFP. Compare GFP^−^ RFP^+^ arrested larva (E and F) with GFP^−^ gravid adults (C and D). Also note that the *paa-1*^+^ RFP-marked array is not generically deleterious for growth as evidenced by the viability of GFP^+^ RFP^+^ adults (E and F). White arrows indicate GFP^−^ worms; white arrowheads indicate GFP^+^ worms; white boxes in (E) and (F) indicate that the image was acquired from a region outside the main panel; yellow boxes indicate regions of increased magnification. Black scale bar in (A) (for A–F), 100 µm; white scale bars in insets (A and E), 20 µm.

To better understand the functions of Nek family kinases during development, we sought to identify extragenic suppressors of molting defects in *nekl-2* and *nekl-3* mutants. Previous studies had identified an allelic series of *nekl-2* and *nekl-3* mutations, including null alleles that arrest at the L1/L2 molt, moderate loss-of-function alleles that arrest as L2/L3s, and weak alleles that are aphenotypic (Yochem *et al.* 2015; Lazetic and Fay 2017). Furthermore, certain combinations of weak alleles of *nekl-2* and *nekl-3* lead to double mutants that display penetrant molting defects and concomitant larval arrest. In the case of strain WY1145, synthetically lethal *nekl-2(fd81)*; *nekl-3(gk894345)* double mutants are maintained by the presence of a rescuing extrachromosomal array (*fdEx286*) that is transmitted to ∼65% of self-progeny, and expresses wild-type *nekl-3* along with a fluorescent reporter (SUR-5::GFP) (Lazetic and Fay 2017). Thus, in this study, WY1145 hermaphrodites gave rise to viable array-containing GFP^+^ progeny as well as array-negative GFP^−^ progeny, 98.8% (*n* = 444) of which arrested as larvae with molting defects (Figure 1A). Rare GFP^−^ escapers that reached adulthood produced progeny that arrested nearly uniformly with molting defects (98.5%, *n* = 1721).

We reasoned that WY1145 could be effective for identifying extragenic suppressors because both *fd81* and *gk894345* are very weak loss-of-function alleles and thus are potentially amenable to genetic suppression, and by carrying out the screen with double mutants, we could obtain suppressors that are specific to either *nekl-2* or *nekl-3*, as well as suppressors of both. As a first approach, we mutagenized WY1145 with EMS and carried out a semiclonal F1 screen (Figure S1). Plates containing candidate suppressor mutations were initially identified based on the increased frequency of F2 GFP^−^ animals reaching adulthood and were subsequently verified by propagating strains derived from isolated F2 GFP^−^ animals for multiple generations. From this screen of ∼8000 haploid genomes, 27 independent isolates were obtained with suppression frequencies ranging from ∼20% to nearly 90% (Figure 1, Table 1, and Table S1). Notably, a pilot screen using a moderate loss-of-function allele, *nekl-3*(*sv3*), alone failed to uncover strong suppressors (data not shown), suggesting that approaches using synthetic-lethal combinations of weak alleles may be particularly productive.

### Use of the peel-1 counterselectable marker to aid suppressor screening

To reduce the labor associated with F1 clonal/semiclonal screening methods, we considered alternative strategies. Because the frequency of naturally occurring GFP^−^ escapers reaching adulthood in WY1145 (∼1–2%) was likely to be much higher than the frequency of authentic suppressors, standard nonclonal approaches were deemed impractical because of the high incidence of false positives. We therefore considered a strategy involving a counterselectable marker to eliminate animals carrying the *nekl-3^+^*–rescuing array. *peel-1* is a sperm-derived toxin that, when overexpressed in *C. elegans* larvae and adults, leads to death at all postembryonic stages (Seidel *et al.* 2008, 2011). We engineered a *nekl-2(fd81)*; *nekl-3(gk894345)* double-mutant strain (WY1255) with a rescuing array containing wild-type *nekl-3*, SUR-5::GFP, and a heat shock–inducible *peel-1* transgene. After incubation of WY1255 at 34° for 2 hr, nearly 100% of array-containing GFP^+^ worms perished within 24 hr. As outlined in Figure S2, WY1255 worms were mutagenized, and 100 individual P0 animals were picked to large plates and incubated at 22° until F2 progeny began to emerge (∼5–6 d). Plates were then heat shocked to kill array-containing animals and were monitored for an additional 7–8 d to identify plates with actively propagating populations of GFP^−^ animals. To prevent the re-emergence of any surviving GFP^+^ worms, plates were heat shocked again after 4 d. From this screen we identified an additional 23 suppressors, demonstrating the utility of this counterselection approach (Table S1). In particular, this method should be useful for identifying suppressors of phenotypes that are <100% penetrant, but it can also be applied to phenotypes that are fully penetrant.

### Development of an SSM to identify suppressors using WGS

Our combined genetic screens identified ∼50 mutants that displayed a wide range of suppressor strengths and included both dominant and recessive alleles (Figure 1, C and D, Table 1, and Table S1, and data not shown). Suppressed strains (*nekl-2*; *nekl-3*; *sup*) were backcrossed to WY1145 and, after preliminary genetic characterization, five strains were selected for further analysis by WGS (Table 1). In addition, genetic studies determined that three of these five suppressors were on LGX, whereas two were autosomal (Table 1 and *Materials and Methods*). The molecular identification of the suppressors, however, presented several technical challenges given that the suppressor mutations did not exhibit obvious phenotypes on their own. In addition, the *nekl-2*(*fd81*) and *nekl-3*(*gk894345*) mutations are aphenotypic and show defects only when combined as double mutants (Lazetic and Fay 2017). Although it was possible to introduce both the *fd81* and *gk894345* mutations into the HA mapping strain (CB4846) using CRISPR/Cas9 methods, as discussed at the beginning of this article, this divergent background has the potential to alter the expression or penetrance of phenotypes. Furthermore, it was unclear if methods that did not make use of the HA strain would provide sufficient mapping resolution of the affected loci to consistently enable facile identification of causal mutations (also see below) (Doitsidou *et al.* 2016).

**Figure 2:**
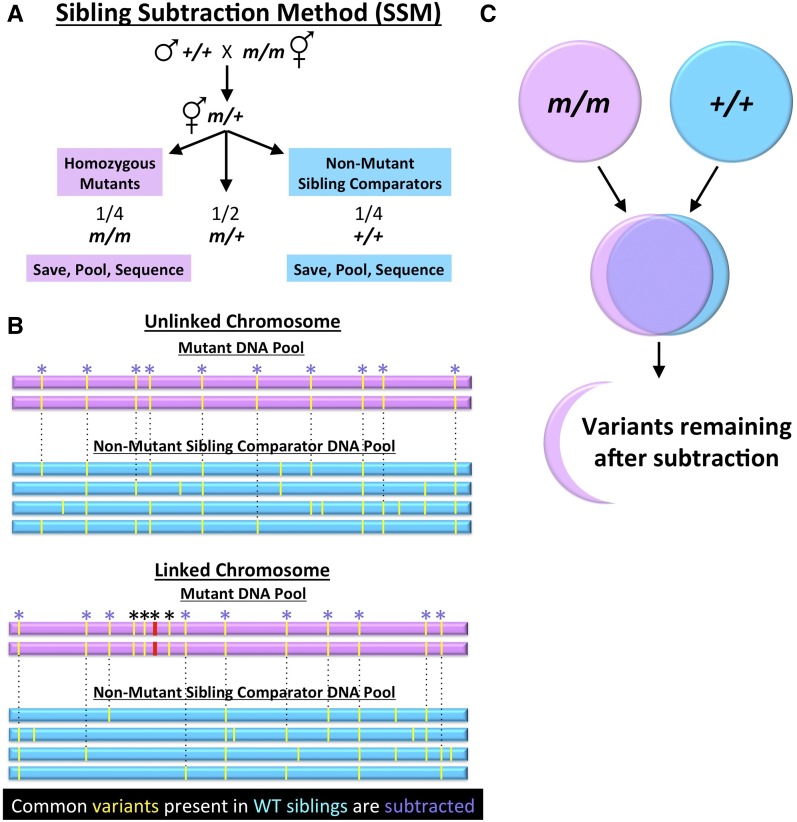
Schematic design of the SSM. (A) The genetic crosses required for generating multiple independent *m/m* and sibling +/+ isolates, which are combined to produce the mutant DNA pool and the nonmutant sibling comparator DNA pool, respectively (also see Figure S3). (B) Simplified representations of sequenced chromosomes from the mutant pool (pink) and nonmutant sibling comparator pool (blue) are shown. Yellow lines indicate variants detected by WGS, and asterisks indicate variants that are homozygous in the mutant pool. Note that the depiction underestimates the true number of variants per chromosome. Dashed lines and purple asterisks indicate variants that are present in both the mutant pool and the nonmutant sibling comparator pool, which can be eliminated (subtracted) as candidate causal mutations. Note that subtraction will remove most or all variants on unlinked chromosomes, whereas homozygous variants very close to the causal mutation (red) may not be subtracted (black asterisks). (C) Venn diagram of subtracted variants (purple) along with the relatively small proportion of remaining candidate variants (pink) after application of the SSM.

We therefore devised a WGS strategy to identify *nekl-2*; *nekl-3* suppressors that did not depend on either variant density mapping or the use of CB4856. We refer to our approach as the SSM, and a generic version of this strategy is shown in Figure 2. Importantly, this strategy can be applied to a variety of phenotypes as well as both simple and complex genetic backgrounds, including mutations that lead to enhancement or suppression. To carry out this approach, *nekl-2(fd81)*; *nekl-3(gk894345)* suppressed strains (*nekl-2*; *nekl-3*; *sup*) were crossed to WY1145 to generate heterozygous (*sup/+*) GFP^+^ F1 cross-progeny, which were picked to individual plates and allowed to self-fertilize (Figure 2A and Figure S3). Next, sibling GFP^+^ F2 progeny from a single F1 parent were picked to individual plates and allowed to produce the F3 generation. Based on standard Mendelian inheritance patterns, a 1:2:1 ratio corresponding to *sup/sup*, *sup/+*, and *+/+* would be expected among the F2 generation. Plates containing F2 parents of the *sup/sup* genotype were recognized based on the high frequency of viable GFP^−^ adult F3s. Moreover, viable GFP^−^ F3 worms from candidate *sup/sup* plates were picked to new plates and allowed to propagate to ensure suppressor homozygosity. Conversely, plates containing parental F2s of the *+/+* genotype were identified by the absence of suppressed (adult GFP^−^) F3 animals (Figure S3). For each suppressor strain, individual *sup/sup* isolates were combined to make genomic DNA, which we refer to as the “suppressed” DNA pool (Figure S3). Corresponding *+/+* isolates were also combined to generate a “nonsuppressed” DNA pool (Figure S3). We note that the number of independent *sup/sup* or *+/+* isolates used to generate the DNA pools ranged from 5 to 15, indicating that as few as five isolates may be sufficient for our approach to be successful.

### Bioinformatic workflow and parameters

**Figure 3:**
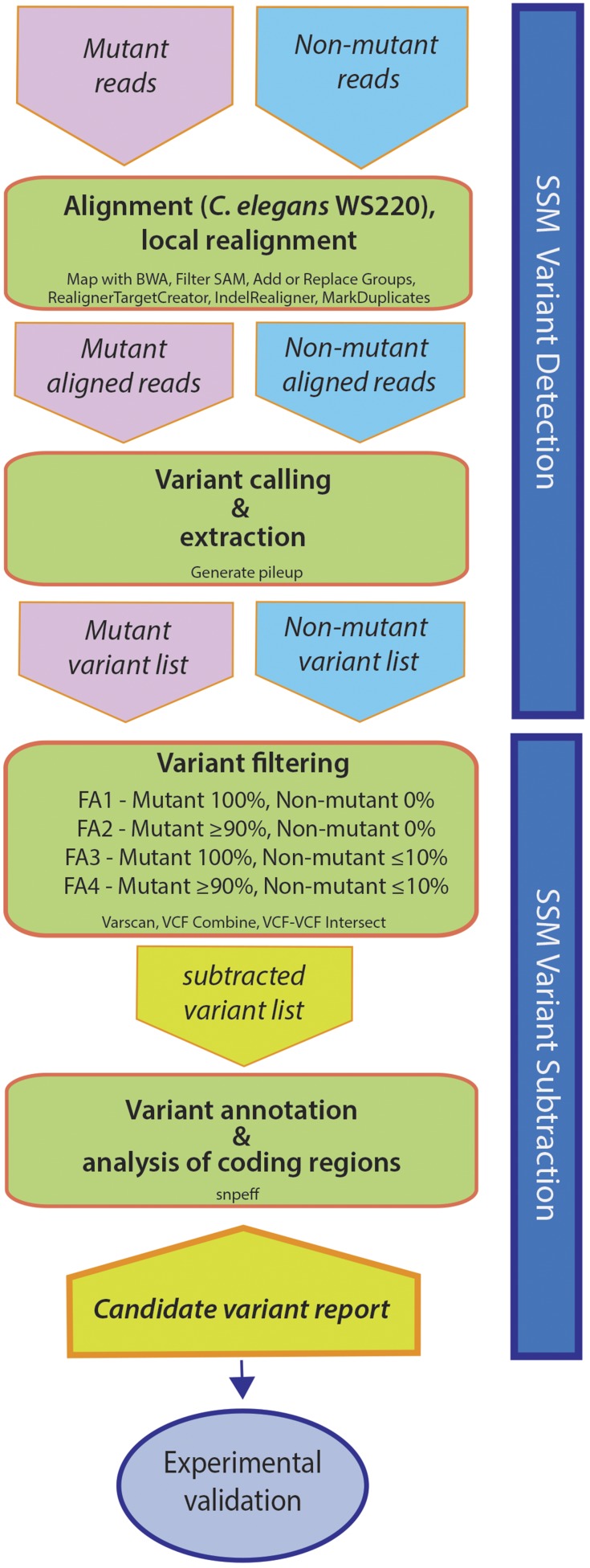
SSM workflow. Overview of the experimental and bioinformatical steps involved in the SSM/WGS method. For additional details, see text and Supplemental Methods, File S1.

The workflow for the experimental and bioinformatic analysis is shown in Figure 3 and is described in detail in the Supplemental Methods in File S1. For our SSM analysis, we tested four different filtering criteria for each of the suppressor strains. This allowed us to determine how different parameters would impact the identification of candidate variants. In our most stringent version (filtering analysis 1; FA1), we required that variants from the suppressed DNA pools be present in 100% of reads, whereas variants from the nonsuppressed sibling DNA pools need only be present in >0% of reads. These parameters therefore minimized the number of candidate variants from the suppressed pools while maximizing the number of variants from the nonsuppressed pools used for subtraction. We predicted that the large majority of strain-specific noncausal mutations acquired before or after mutagenesis would therefore be subtracted, leaving a manageable number of candidates requiring experimental validation (Figure 2, B and C).

In practice, we found that FA1 eliminated 98.4% (range, 98.0–99.0%) of all variants detected in the five suppressed DNA pools (Table 2). Likewise, when considering variants leading to changes in protein structure (nonsense, missense, frameshift, and splice site mutations), an average of 98.8% (range, 98.2–100%) of variants were subtracted. Manual examination of candidate variants following computational subtraction led to an additional reduction in the total number or candidate variants for all five strains from 13 to 10 (see File S2). In two of these cases, the subtraction of a common variant failed because the required number of nonsuppressed sibling reads fell below our set computational threshold of three. Nevertheless, an examination of the two high-quality reads available from each strain indicated that the variants were present in both suppressed and nonsuppressed strains and thus could be eliminated as causal.

**Table 2:** Summary of SSM/WGS data

Filtering Analysis (FA1–4)	Strain	Number of Variants Before SSM			Number of Variants After SSM		
		Suppressed DNA Reads		Nonsuppressed Reads	Total	Change in Coding*^a^*	Change in Coding Manually Filtered*^a^*^,^*^b^*
		Total	Change in Coding*^a^*	Total			
FA1 100%^Sup^ >0%^Non-Sup^	WY1208	2018	210	424,656	31	4	4
	WY1209	2075	222	471,857	42	4	2
	WY1210	2096	230	1,021,954	37	3	2
	WY1211	1898	218	860,417	19	2	2
	WY1217	2063	208	969,292	36	0	0
FA2 ≥90%^Sup^ >0%^Non-Sup^	WY1208	2652 (+31.4%)	245	424,656	42 (+35.5%)	4	4
	WY1209	2845 (+37.1%)	260	471,857	45 (+7.1%)	4	2
	WY1210	2732 (+30.3%)	258	1,021,954	46 (+24.3%)	6	5
	WY1211	2347 (+23.7%)	237	860,417	29 (+52.6%)	5	5
	WY1217	2734 (+32.5.%)	239	969,292	41 (+13.9%)	1	1
FA3 100%^Sup^ ≥10%^Non-Sup^	WY1208	2018	210	97,520 (−77.0%)	38 (+22.6%)	5	5
	WY1209	2075	222	117,834 (−75.0%)	44 (+4.8%)	5	3
	WY1210	1898	230	74,586 (−92.6%)	44 (+18.9%)	3	2
	WY1211	2063	218	91,174 (−89.4%)	20 (+5.3%)	2	2
	WY1217	1891	208	79,897 (−91.8%)	37 (+2.8%)	0	0
FA4 ≥90%^Sup^ ≥10%^Non-Sup^	WY1208	2652	245	97,520	52 (+64.5%)	5	5
	WY1209	2845	260	117,834	47 (+21.6%)	5	3
	WY1210	2732	258	74,586	56 (+40.5%)	6	5
	WY1211	2347	237	91,174	30 (+59.3%)	5	5
	WY1217	2734	239	79,897	43 (+30.1%)	1	1

Sequencing was carried out on pooled independent isolates from suppressed and nonsuppressed strains as described in *Materials and Methods*. Numbers in parentheses in indicate positive (+) or negative (−) percentage changes for each strain relative to FA1. For more information see Supplemental Methods, File S1.

a Includes nonsense, missense, frameshift, and splice site mutations.

b For specific details on manual filtering see File S1.

Although effective in limiting the number of candidate causal variants, it was possible that our most stringent parameters (FA1) could, however, lead to the occurrence of false negatives. Consistent with this possibility, FA1 failed to identify coding change candidates for strain WY1217, suggesting that at least one true positive may have been eliminated in our analysis (Table 2). One source of false negatives could be due to sequencing errors or misalignments, which could lead to a failure to call an authentic variant in the suppressed DNA pool. False negatives could also result if one of the worm isolates used for DNA sequencing was incorrectly assigned at the level of phenotype. For example, it is possible that DNA pools for dominant alleles could contain animals that were heterozygous, thus reducing the percentage of causal variant reads in the suppressed DNA pool to <100%. Conversely, in the case of weakly penetrant recessive alleles, heterozygous strains could be accidentally assigned to the nonsuppressed pool, thereby increasing the percentage of causal variant reads to >0% and leading to their subtraction. Finally, regardless of the above filtering criteria, a false negative would occur if the causal mutation were to be located in a noncoding region (also see below).

We therefore analyzed sequencing data from all our strains using three additional filtering criteria (FA2–4). In the case of FA2, we reduced the requirement for variant calls from the suppressed DNA pools from 100 to ≥90%, while maintaining the requirement for nonsuppressed variant reads at >0% (File S2 and Table 2). Compared to FA1, this increased the number of called variants in the suppressed DNA pools by an average of 31.0% (range, 23.7–37.1%), increased the average number of variants after subtraction by 18.7% (range, 7.1–52.6%), and raised the total number of coding-change variants (for all five strains) after subtraction from 13 to 20. Notably, FA2 parameters led to the identification of a single coding-change candidate for strain WY1217 (File S2 and Table 2). Further examination of this candidate revealed that a single read from the suppressed DNA pool had been improperly aligned with BWA, leading to its omission in FA1.

For FA3, we required variants from the suppressed DNA pools to be present in 100% of reads but mandated that variants from the nonsuppressed DNA pools be present at ≥10%, thereby reducing the number of variants used for subtraction by an average of 87.8% (range, 75.0–92.6%). This led to an average increase in the number of candidate variants following subtraction by 8.1% (range, 2.8–22.6%) relative to FA1, and increased the total number of coding-change variants from 13 to 15 (File S2 and Table 2). Finally, by requiring variants to be present in ≥90% of reads in the suppressed DNA pools and ≥10% in the nonsuppressed DNA pools (FA4), we observed an average increase over FA1 in the number of variants following subtraction by 27.6% (range, 21.6–64.5%) along with an increase in total coding-change variants from 13 to 22. We note that even under these more liberal filtering criteria, an average of 98.2% of variants were subtracted using SSM. In summary, by using less stringent filtering criteria (FA2–FA4), we observed a modest increase in the number of candidate causal variants and, in the case of FA2 and FA4, identified a single coding-change candidate for strain WY1217.

### Experimental validation of variants identified by the SSM

Having identified candidate casual variants, we next sought to validate the identities of all four recessive causal mutations using a combination of RNAi, transgenic rescue experiments, and CRISPR/Cas9. In the case of strains WY1208, WY1209, and WY1211, we focused on candidates obtained using FA1, whereas for strain WY1217, FA2 criteria were used (File S2 and Table 2). A summary of the findings and methods used for validation is shown in Table 3. One example is strain WY1211 (*nekl-2*; *nekl-3*; *fd134*), which exhibits 34% suppression (*n* = 199), making it the weakest of the suppressors subjected to SSM/WGS (compare Figure 1, A and B to Figure 1, C and D). After subtraction using the most stringent parameters (FA1), only two candidate variants remained that affected coding regions, corresponding to missense mutations in C27F2.9 and F48E8.5/*paa-1* (Table 1). Notably, both variants reside within a 0.6-map unit-long region on the left arm of LGIII. C27F2.9 encodes a protein with homology to JMJD8, and the detected variant leads to a R220C substitution in a residue that is not highly conserved among closely related species. *paa-1* encodes an essential gene that is orthologous to the PR65 structural subunit of the mammalian PP2A protein phosphatase. Moreover, the identified variant in *paa-1* causes a G550E substitution in a glycine residue that is highly conserved throughout metazoans (data not shown). Two lines of evidence demonstrated that the *fd134* causal mutation corresponds to the lesion in *paa-1*. First, we recreated the identical (G→A) mutation using CRISPR/Cas9 methods in the WY1145 background and obtained two independent suppressed lines. Second, we injected fosmids encoding the wild-type *paa-1* locus into WY1211 and observed clear desuppression in three of three lines (Figure 1, E and F). Our identification of *paa-1* as a suppressor of *nekl-2*; *nekl-3* defects suggests that the *C. elegans* PP2A complex may oppose NEKL-2/3 kinase activity, possibly by dephosphorylating NEKL2/3 targets. Likewise the causal mutations for strains WY1208, WY1209, and WY1217 were successfully identified using a minimum of two independent methods (Table 3).

**Table 3:** Confirmation of recessive suppressor mutations

Strain/Allele	Candidate Gene	Molecular Lesion	RNAi Phenocopy*^a^*	CRISPR/Cas9 Phenocopy*^b^*	Transgenic Rescue*^c^*
WY1208 *fd130*	C04A11.4	G/A	N.D.	+	+
		13692151 X		*n* = 3	*P* < 0.0001
		W494Stop			
WY1209 *fd131*	F56D12.6	C/T	+	+	N.D.
		1320072 II	*P* < 0.0001	*n* = 2	
		Q904Stop			
WY1211 *fd133*	F48E8.5	G/A	N.D.	+	+
		5452501 III		*n* = 2	*P* < 0.0001
		G550E			
WY1217 *fd139*	B0302.1	5 bp del.	+	+	+
		17191744 X	*P* < 0.0001	*n* = 3	*P* < 0.0001
		S989F.S.			

For additional details on RNAi, CRISPR/Cas9, and transgenic rescue studies, see *Materials and Methods*, File S1 and Table S2. F.S. indicates frameshift. *P* values were derived for proportions using the N-1 chi-squared test.

a “+” indicates that statistically significant suppression was observed following injection of WY1145 with corresponding dsRNAs.

b “+” indicates that suppression of starting strain WY1145 was observed following editing of the corresponding loci. “N” indicates the number of suppressed independent CRISPR/Cas9-generated lines that were sequence verified.

c “+” indicates that statistically significant rescue was observed following injection of corresponding suppressed strains with fosmids encoding the candidate loci. Note that in the case of suppressor mutations, rescued animals are no longer suppressed and are molting defective.

In the case of the dominant suppressor, *fd132* (WY1210), FA1 criteria identified coding variants in two genes, T09B9.4 (amino acid substitution M399I) and W07E11.1 (amino acid substitution P1181L). Notably, both genes are located on LGX, which genetic mapping had shown to harbor the causal mutation, and are positioned within ∼300,000 bp of each other (∼0.25 map units). Moreover, both genes reside within a 1.7 Mb region that contains 13 consecutive variants that are present in 100% of suppressed DNA reads and 0% of the nonsuppressed. Genomic engineering of the T09B9.4 and W07E11.1 mutations using CRISPR/Cas9, however, did not lead to genetic suppression, indicating that neither mutation likely corresponds to the causative change in this strain. Consistent with this, RNAi of both genes failed to revert suppression of WY1210, which could be expected if *fd132* was a gain-of-function allele, or to induce suppression in strain WY1145, which might be anticipated if *fd132* was a dominant negative allele. We next examined coding variants in three additional genes (F53A9.7, T05A10.1, and R04E5.10) that were detected using FA2/4 criteria (Table 2). All three genes are on LGX and closely flank the 1.7 Mb region containing T09B9.4 and W07E11.1. However, precise editing of these genes using CRISPR/Cas9 failed to produce genetic suppression. Thus, the *fd132* causal mutation may reside in a noncoding region, such as an element that promotes transcriptional or posttranscriptional repression, or it is possible WGS/SSM may have failed to identify the causal coding variant. However, we note that the 1.7 Mb region, which also corresponds to the region implicated by EMS density mapping (see below), does not contain any sequencing gaps within coding regions, further implicating noncoding elements.

### Comparison of SSM and EMS density mapping methods

**Figure 4:**
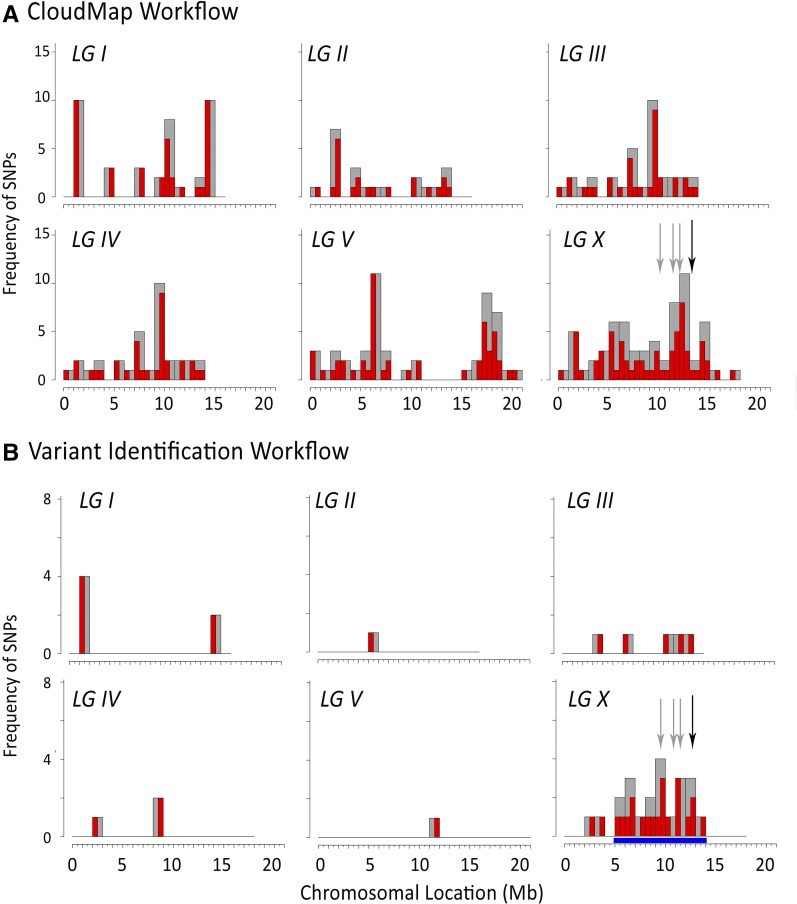
EMS density mapping comparison in WY1208. EMS density mapping for the five suppressor strains was carried out using the CloudMap workflow on Galaxy (A) or using our variant identification workflow based on allele frequencies (B). The *x* axis indicates the location on each chromosome in megabases (Mb). The number of EMS signature SNPs are indicated on the *y* axis; red bars indicate a 0.5-Mb region, and gray bars indicate a 1.0-Mb region. Black arrows indicate the location of the identified causal mutations, and gray arrows indicate noncausal mutations identified by SSM. The blue bar in (B) indicates the implicated region on LGX that contained five coding-change candidates.

We next compared our subtraction approach to the EMS density mapping method, which identifies regions of the genome harboring high frequencies of EMS signature variants that are maintained following serial backcrossing (Zuryn *et al.* 2010). Importantly, use of SSM does not preclude EMS density mapping methods and these methods can be viewed as complementary. We first obtained EMS density maps for strains WY1208, WY1209, WY1210, WY1211, and WY1217 using the published CloudMap workflow, which is available on the Galaxy web platform (Minevich *et al.* 2012). In each case, we subtracted called variants that were present in any of the other suppressed strains. Although a subset of the major peaks generated by EMS density mapping were consistent with the locations of our identified causal mutations; in most cases, this analysis failed to provide unambiguous localization of our causal mutations to either chromosomes or to chromosomal subregions (Figure 4 and Figure S4).

Because the EMS density mapping workflow on CloudMap uses Bayesian methods to call variants, which we found to lead to some false positives in our analysis, we were next interested to perform EMS density mapping using our workflow, which bases variant calls on absolute allele frequencies (*e.g.*, >0–100%). Once again, we subtracted variants that were present in any of the other suppressed strains before generating density maps. Notably, this approach greatly reduced the total number of EMS variants and dramatically improved the clarity of the mapping method (Figure 4 and Figure S5). For example, in the case of WY1208 this resolved the chromosomal location of *fd130* to a region on LGX (5–14 Mb) that contained five homozygous coding-change candidates, including C04A11.4. Likewise, candidates for WY1209 (4), WY1210 (3), and WY1211 (2) were identified, although in the case of WY1217 no coding-change candidates were identified under the major peak on LGX (Figure S5).

When carrying out EMS density mapping, it is theoretically advantageous to sequence multiple mutant strains derived from the same parent as this increases the number of background variants that can be identified and subtracted before generating density maps. To test this directly, we generated EMS density maps for WY1208 using WY1211 only as our subtraction strain. Notably, this led to considerably poorer resolution of the causal candidate to either a chromosome or chromosomal subregion; eight coding-change candidates were implicated under the major peak on LGX (2.5–14 Mb) (Figure S6). In summary, when applying our variant identification workflow, SSM generally outperformed EMS density mapping and may be particularly advantageous in situations where a limited number of strains are to be sequenced. However, additional studies will be necessary to determine the relative advantages or disadvantages of SSM and other available methods.

## Discussion

We have demonstrated SSM/WGS to be a simple and effective approach for identifying causal mutations in *C. elegans*. Importantly, this approach does not require the use of polymorphic strains (*e.g.*, CB4856) and does not rely on variant mapping methods. In addition, SSM should be generally applicable to other animal systems. SSM can be made flexible in its use of different filtering parameters (FA1–4; Table 2), which can allow for a progressive interrogation in the search for causal candidate variants.

Based on our study, SSM appears to provide somewhat better resolution of candidates than EMS density mapping methods alone, particularly in cases where fewer total strains are subjected to sequencing (Figure 4, Figure S4, Figure S5, and Figure S6). We note that although the majority of published studies using EMS density mapping do not directly state the number of causal candidates obtained using this approach, Zuryn and colleagues reported two, four, and nine candidates for the three test strains examined (Zuryn *et al.* 2010). Importantly, EMS density mapping can be used in a complementary fashion with SSM, and altering the published workflow (*e.g.*, calling variants based on absolute counts rather than probabilities) may provide increased resolution over the current version available through CloudMap, as implemented on Galaxy.

One drawback to SSM is the additional costs of having to sequence both mutant and nonmutant sibling comparator samples. This, however, must be balanced by the potential for reducing the number of causal candidates, which can be time consuming and expensive to validate. Another drawback of SSM relative to high-density mapping methods, such as the use of CB4856 or VDM, is that noncoding causal variants may be more difficult to identify using SSM. This is because the reduced mapping resolution provides less guidance regarding which variants should be prioritized for initial validation tests. Finally, we note that our average coverage for the five genomes was 20–30×, and it is possible that a somewhat higher coverage level would provide better information. Nevertheless, our current coverage did allow for the identification of four out of four recessive alleles from our screen. Our approach did not allow for a direct comparison between SSM and “bulked segregant” VDM methods; future parallel assessments of these methods may prove useful.

In addition, we showed that a counterselection method using an extrachromosomal array encoding the inducible toxin *peel-1* can provide a highly efficient means for identifying genetic suppressors, especially in cases of incomplete penetrance of the starting strain. Our studies also underscore the utility of using weak alleles in synthetic-lethal combinations as starting strains for suppressor screens. In particular, recent advances in genomic engineering (Arribere *et al.* 2014; Paix *et al.* 2014, 2015; Dickinson and Goldstein 2016), as well as the Million Mutation resource (Thompson *et al.* 2013), make the identification of weak alleles and combinatorial-dependent phenotypes highly feasible. Finally, because SSM/WGS is not affected by genetic background, it should be possible to identify genetic modifiers of complex genotypes in a straightforward manner.

## 

## Supplementary Material

Supplemental material is available online at www.g3journal.org/lookup/suppl/doi:10.1534/g3.117.300135/-/DC1.
