# Supplementary figures and images for: Use of a Sibling Subtraction Method for Identifying Causal Mutations in Caenorhabditis elegans by Whole-Genome Sequencing
Source: G3 (Bethesda). 2017 Dec 12;8(2):669–78. doi: 10.1534/g3.117.300135 (PMC5919755; doi:10.1534/g3.117.300135)

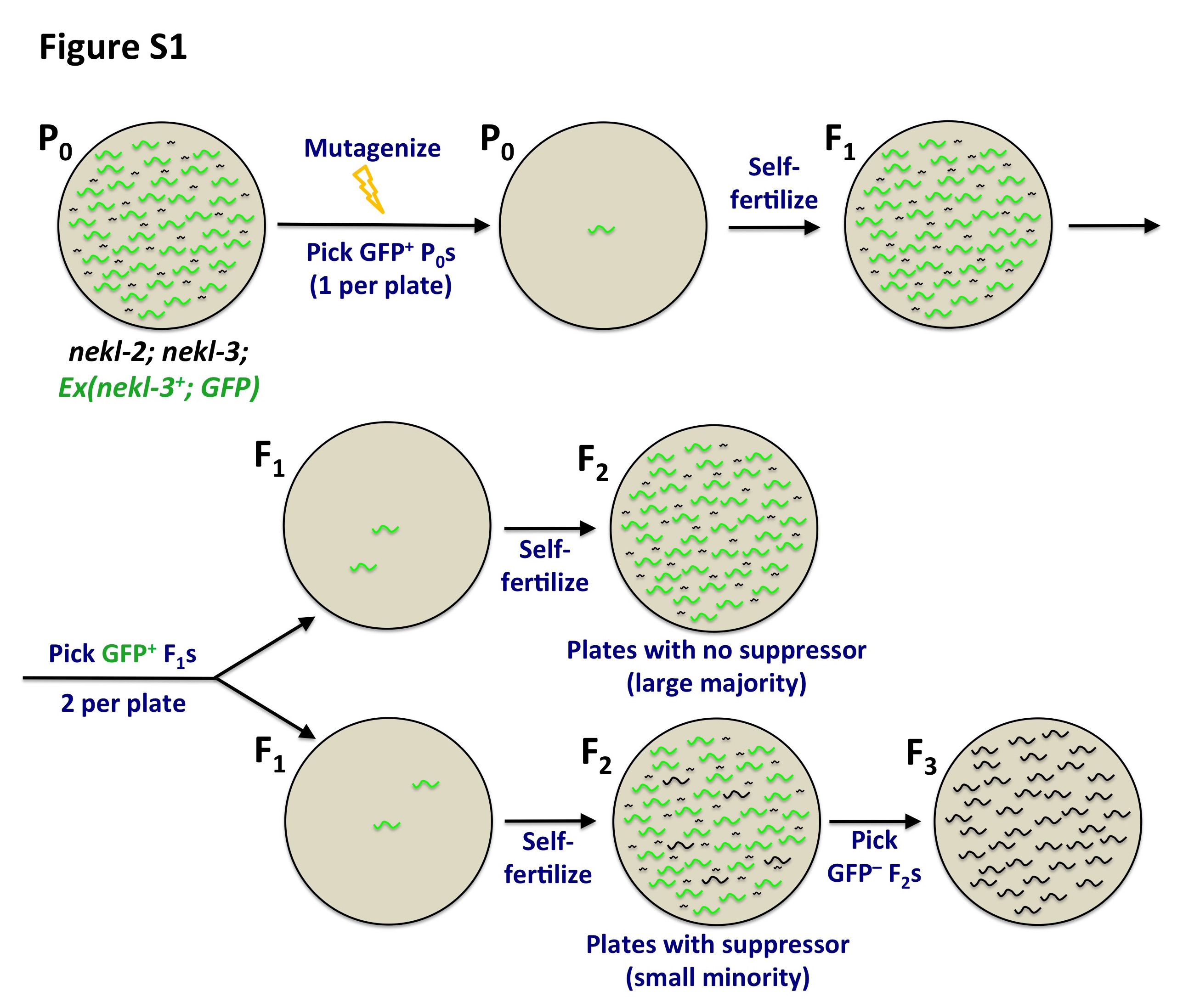

Supplement: Supplementary file 1 [file 669FigureS1.jpg]

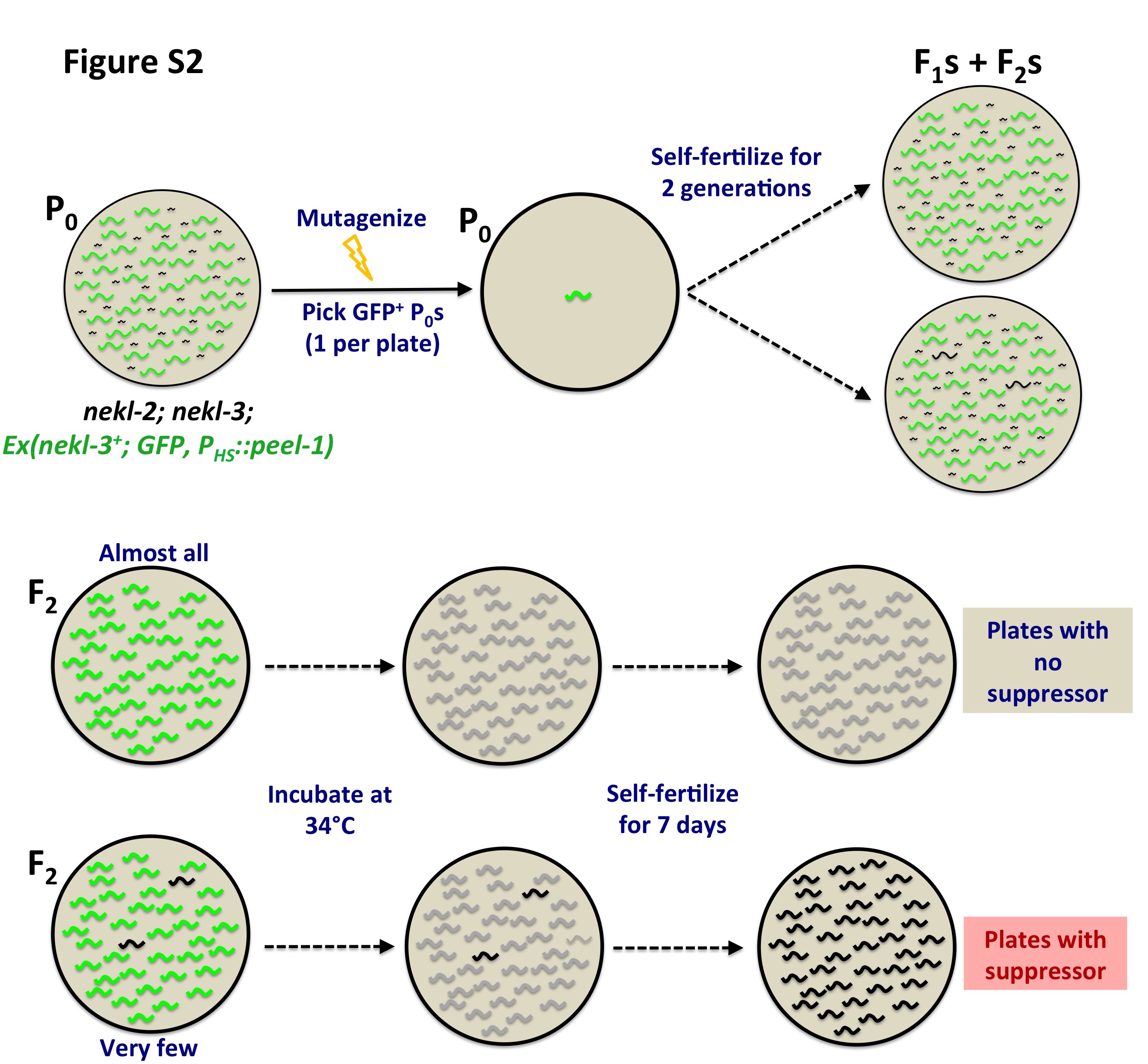

Supplement: Supplementary file 2 [file 669FigureS2.jpg]

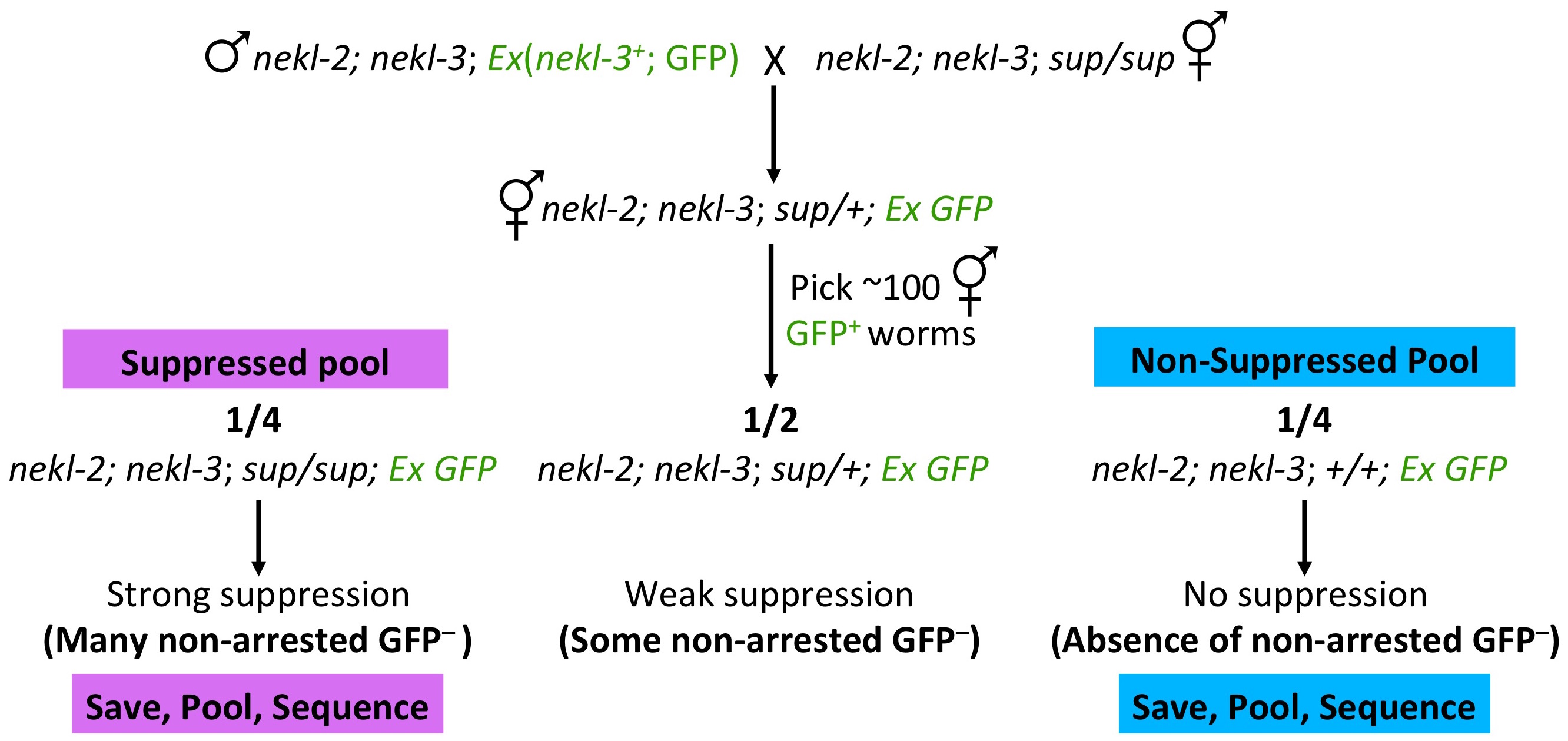

Supplement: Supplementary file 3 [file 669FigureS3.jpg]

CloudMap Workflow

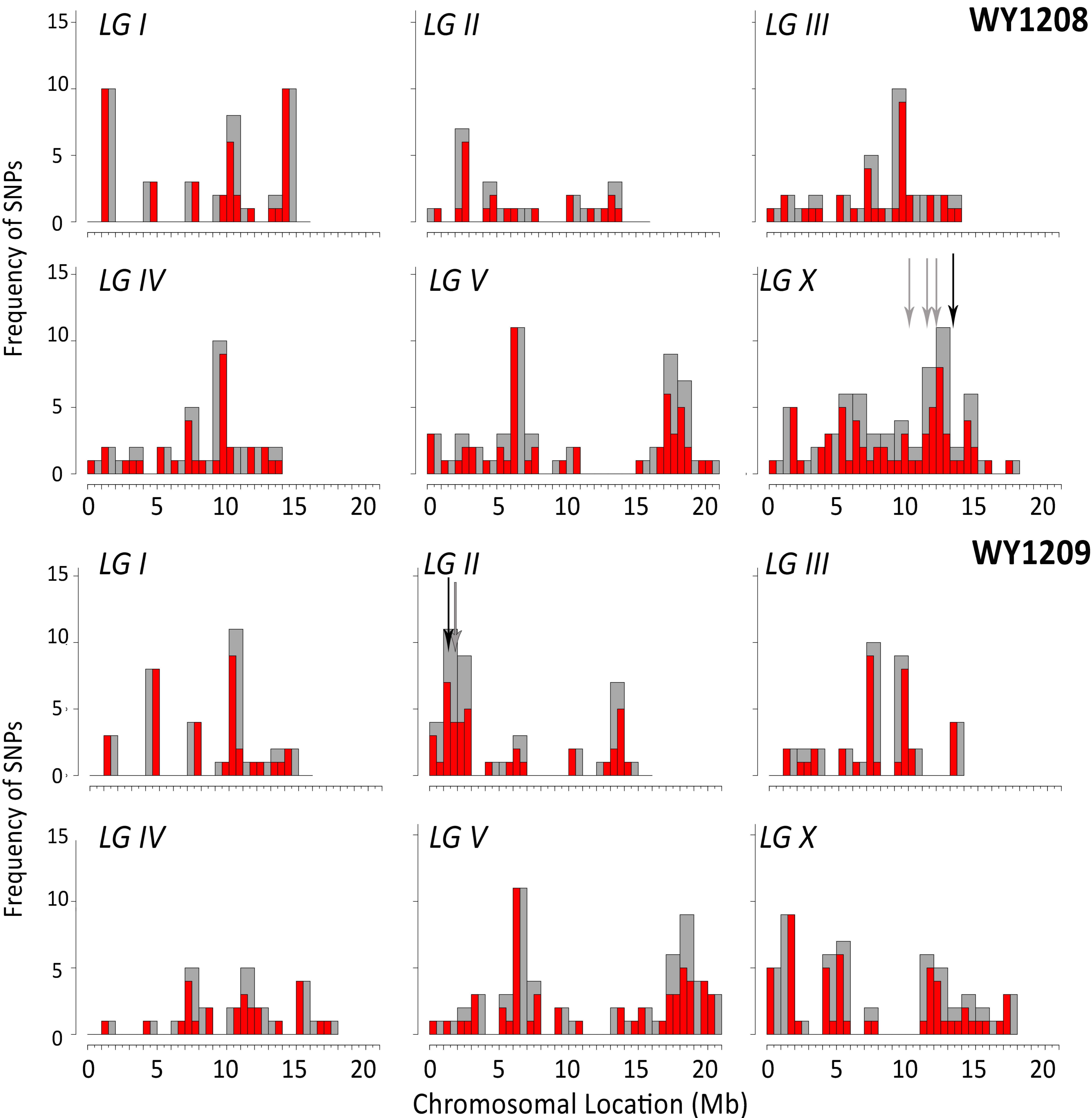

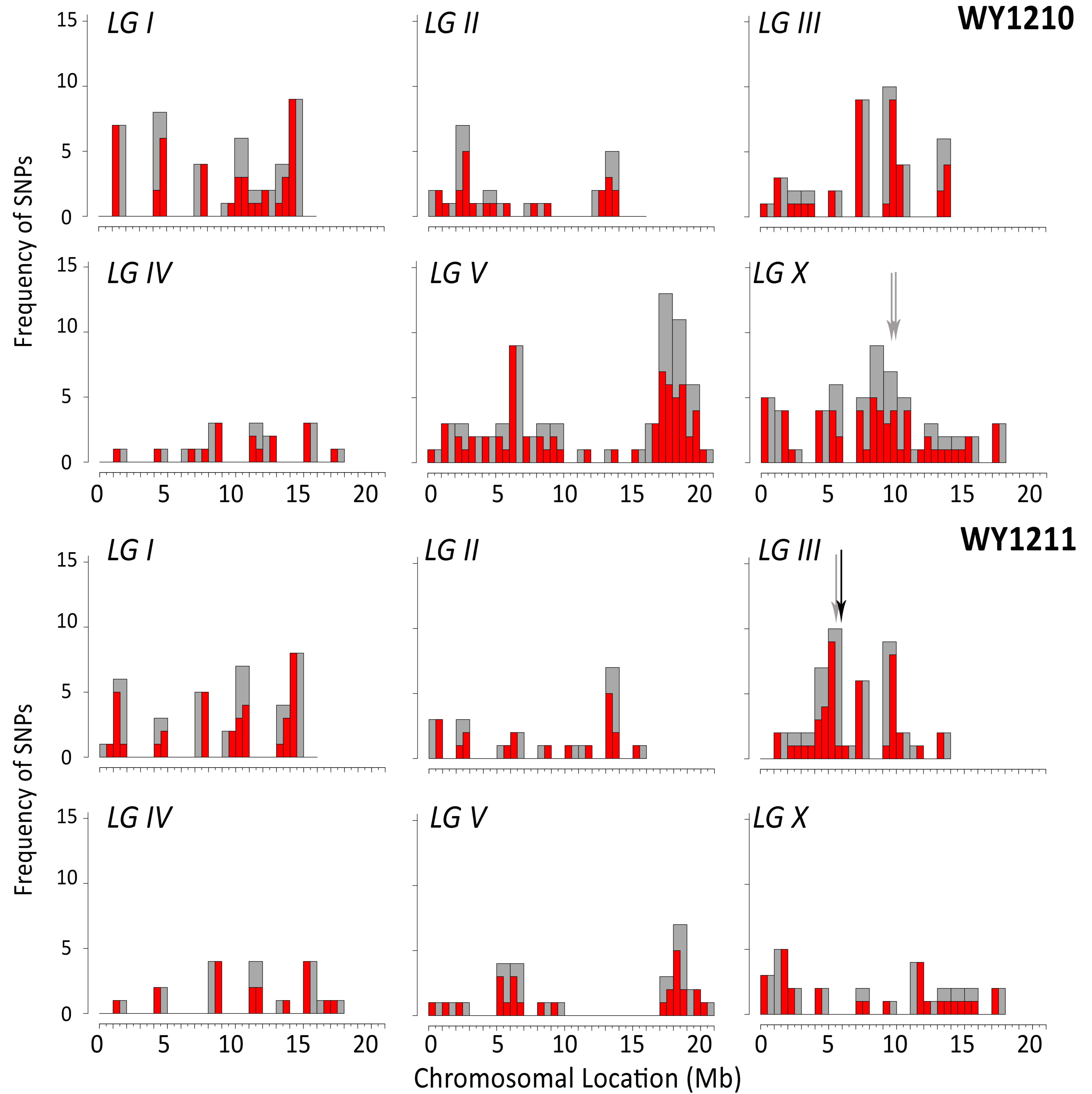

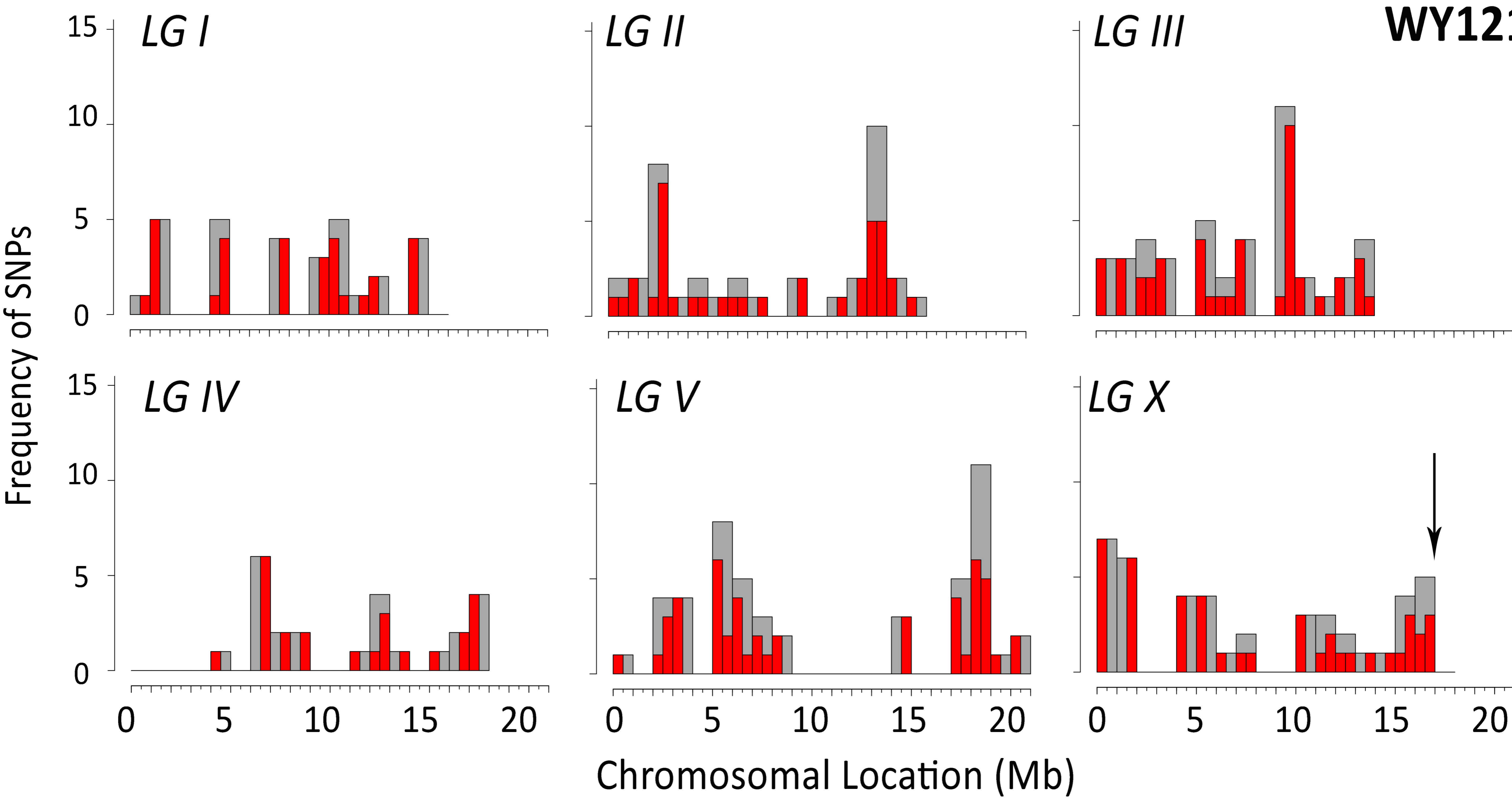

Supplement: Supplementary file 4 [file 669FigureS4.pdf]

# Variant Identification Workflow

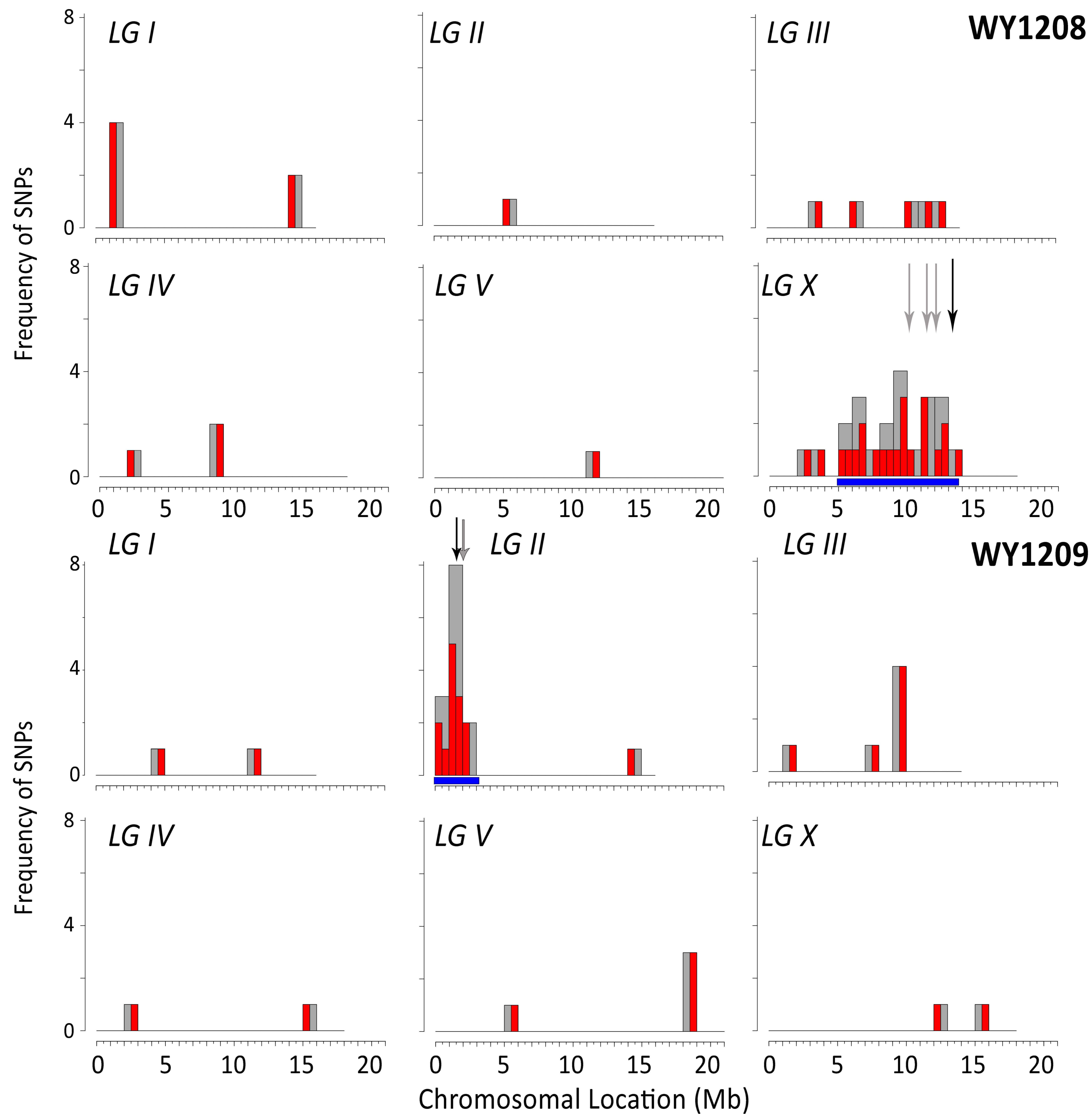

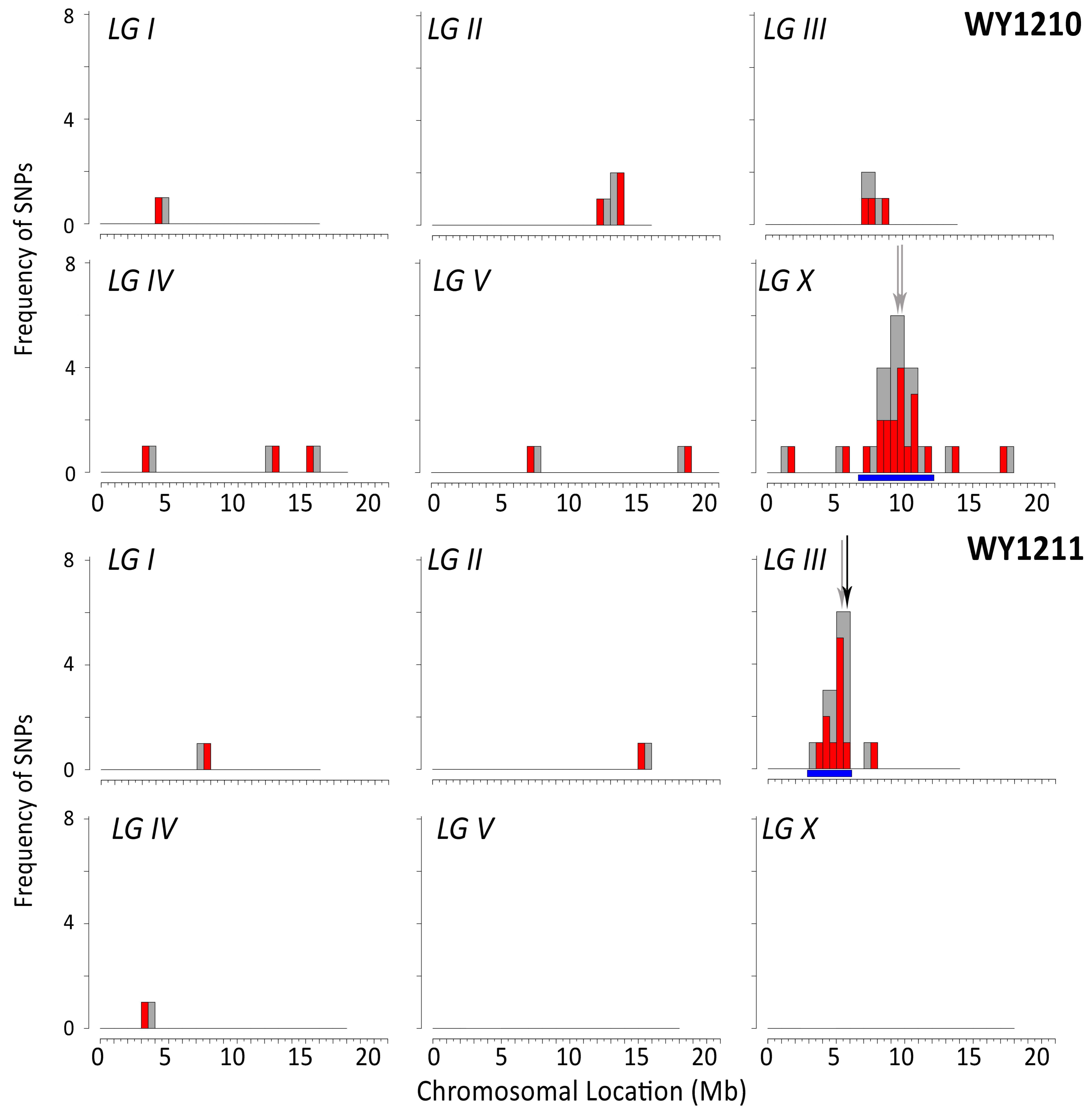

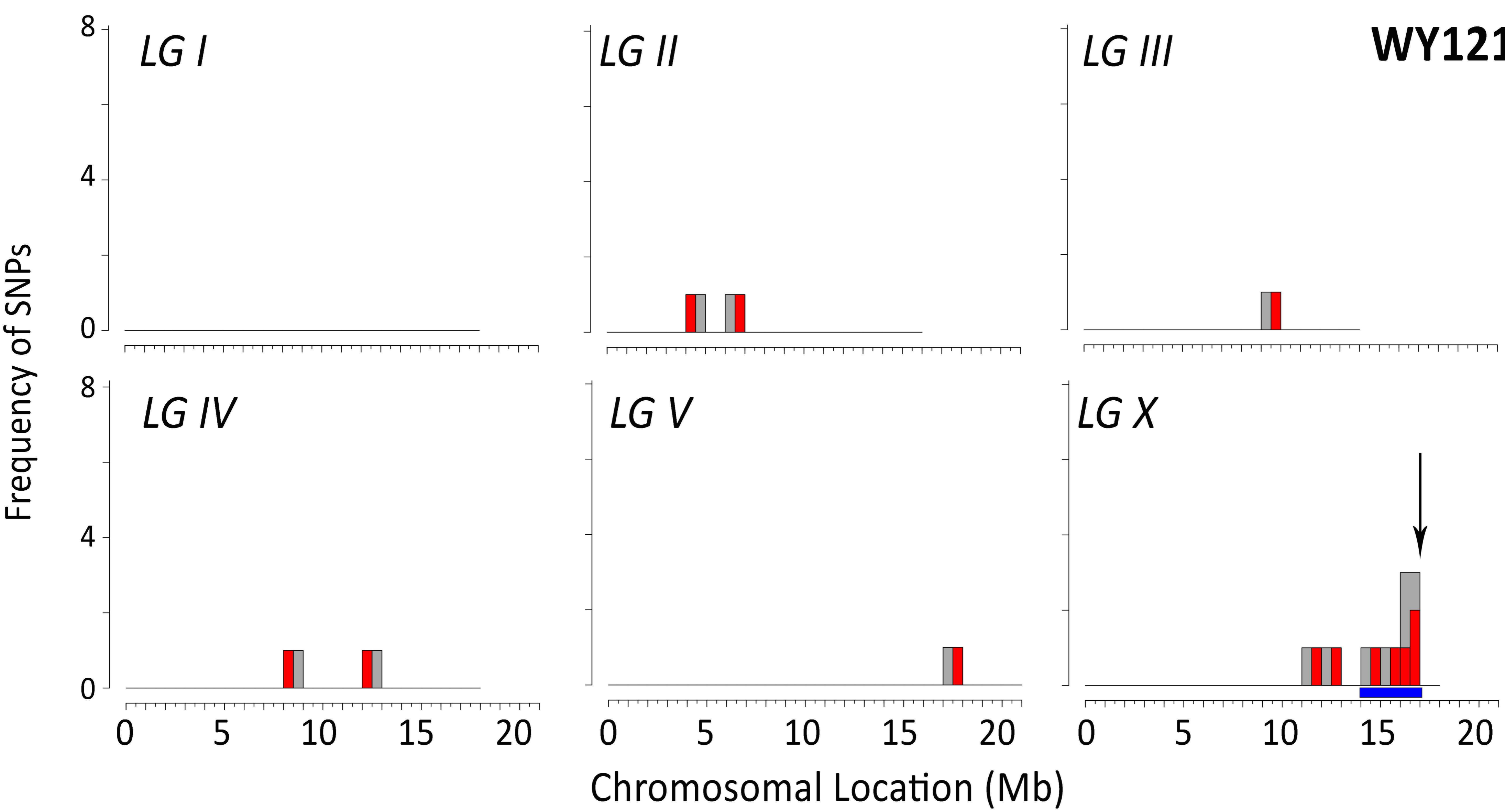

Supplement: Supplementary file 5 [file 669FigureS5.pdf]

# Single comparator analysis: Variant Identification Workflow

**WY1208**

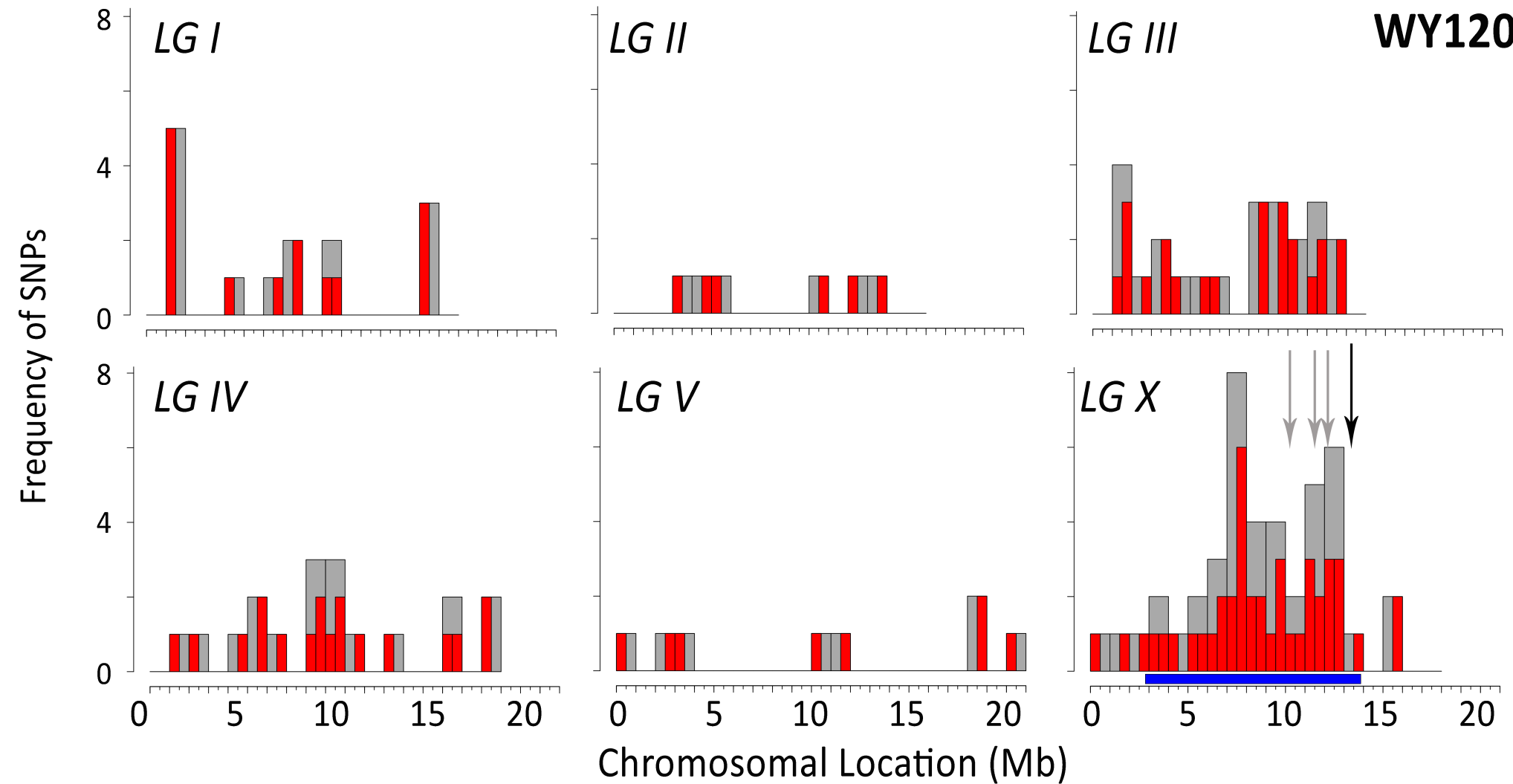

Supplement: Supplementary file 6 [file 669FigureS6.pdf]
