# Supplementary material for: Use of a Sibling Subtraction Method for Identifying Causal Mutations in Caenorhabditis elegans by Whole-Genome Sequencing
Source: G3 (Bethesda). 2017 Dec 12;8(2):669–78. doi: 10.1534/g3.117.300135 (PMC5919755; doi:10.1534/g3.117.300135)
Supplement: Supplementary file 7 [file 669TableS1.pdf]

Table S1. List of *nekl-2; nekl-3* suppressors.

Strains isolated from F1 semi-clonal screen				
Strain Name	Allele	% Suppression (n)	Dominant/Recessive	Autosome/LGX
Strains subjected to SSM & WGS				
WY1208	*fd130*	83 (223)	Recessive	LGX
WY1209	*fd131*	87 (360)	Recessive	Autosome
WY1210	*fd132*	50 (276)	Dominant	LGX
WY1211	*fd133*	35 (422)	Recessive	Autosome
WY1217	*fd139*	76 (235)	Recessive	LGX
Strains NOT subjected to SSM & WGS				
WY1212	*fd134*	34 (387)	Weak Dominant	N.D.
WY1213	*fd135*	N.D.	N.D.	N.D.
WY1214	*fd136*	25 (374)	Weak Dominant	N.D.
WY1215	*fd137*	35 (277)	Weak Dominant	N.D.
WY1216	*fd138*	48 (315)	N.D.	N.D.
WY1218	*fd140*	47 (430)	N.D.	N.D.
WY1220	*fd142*	37 (138)	N.D.	N.D.
WY1221	*fd143*	33 (425)	N.D.	N.D.
WY1222	*fd144*	33 (167)	N.D.	N.D.
WY1223	*fd145*	32 (613)	N.D.	N.D.
WY1224	*fd146*	31 (30)	N.D.	N.D.
WY1225	*fd147*	30 (389)	N.D.	N.D.
WY1267	*fd151*	63 (300)	Recessive	Autosome
WY1268	*fd152*	55 (77)	N.D.	N.D.
WY1269	*fd153*	79 (333)	N.D.	N.D.
WY1270	*fd154*	51 (206)	Recessive	Autosome
WY1271	*fd155*	94 (306)	Recessive	LGX
WY1272	*fd156*	N.D.	N.D.	N.D.
WY1273	*fd157*	N.D.	N.D.	N.D.
WY1274	*fd158*	N.D.	N.D.	N.D.
WY1275	*fd159*	N.D.	N.D.	N.D.
WY1276	*fd160*	N.D.	N.D.	N.D.
Strains isolated from counter-selection screen				
Strains NOT subjected to SSM & WGS				
WY1277	*fd161*	63 (260)	N.D.	N.D.
WY1278	*fd162*	78 (639)	Recessive	Autosome
WY1279	*fd163*	83 (121)	N.D.	N.D.
WY1280	*fd164*	N.D.	N.D.	N.D.
WY1281	*fd165*	57 (130)	N.D.	N.D.
WY1282	*fd166*	34 (938)	Recessive	Autosome
WY1283	*fd167*	71 (219)	N.D.	N.D.
WY1284	*fd168*	N.D.	N.D.	N.D.

WY1285	*fd169*	86 (179)	N.D.	N.D.
WY1286	*fd170*	43 (112)	Recessive	Autosome
WY1287	*fd171*	77 (220)	N.D.	N.D.
WY1288	*fd172*	74 (282)	N.D.	N.D.
WY1289	*fd173*	13 (643)	Recessive	Autosome
WY1290	*fd174*	77 (137)	N.D.	N.D.
WY1291	*fd175*	85 (66)	N.D.	N.D.
WY1292	*fd176*	78 (201)	N.D.	N.D.
WY1293	*fd177*	N.D.	N.D.	N.D.
WY1294	*fd178*	46 (190)	N.D.	N.D.
WY1295	*fd179*	84 (266)	N.D.	N.D.
WY1296	*fd180*	76 (223)	N.D.	N.D.
WY1297	*fd181*	80 (93)	N.D.	N.D.
WY1298	*fd182*	30 (123)	Recessive	Autosome
WY1299	*fd183*	N.D.	N.D.	N.D.
