# Supplementary material for: Use of a Sibling Subtraction Method for Identifying Causal Mutations in Caenorhabditis elegans by Whole-Genome Sequencing
Source: G3 (Bethesda). 2017 Dec 12;8(2):669–78. doi: 10.1534/g3.117.300135 (PMC5919755; doi:10.1534/g3.117.300135)
Supplement: Supplementary file 8 [file 669TableS2.pdf]

Alleles	CRISPR/Cas9 Modified Regions
**Wild type**	(1419) GGAAAATGAGTTCTGTTACGAAGGTGGCTGTGGTAGTCGAAACGATCAATG
fd208	(1419) GGAAAATGAGTTCTGTTACG_AGGTGGCTGTGGTAGTCGAAACGATCAATG
**Wild type**	(1419) GGAAAATGAGTTCTGTTACGAAGGTGGCTGTGGTAGTCGAAACGATCAATG
fd209	(1419) GGAAAATGAGTTCTGTTAC__AGGTGGCTGTGGTAGTCGAAACGATCAATG
**Wild type**	(1419) GGAAAATGAGTTCTGTTACGAAGGTGGCTGTGGTAGTCGAAACGATCAATG
fd210	(1419) GGAAAATGA_____________GGTGGCTGTGGTAGTCGAAACGATCAATG
**Wild type**	(2907) GTAAGCTTGGAAAT_GCTCGATCGGAAGCCGGCGCAGGTCTTCGTGCAATT
fd211	(2907) GTAAGCTTGGAAATTGC___A_CG_AAGAC_________CTTCGTGCAATT
**Wild type**	(2907) GTAAGCTTGGAAATGCTCGATCGGAAGCCGGCGCAGGTCTTCGTGCAATTT
fd212	(2907) GTAAGCTTGGAAATGCTCGATCGG__________AGGTCTTCGTGCAATTT
**Wild type**	(1620) CAACGCCGCAAAGAGTTTGAAGCGAATTGGAAAGAACTTGACCCCAAGTAC
fd216	(1620) CAACGCCGCAAAGAGTTTAAAGCGAATTGAAAAGAACTTGACCCCAAGTAC
**Wild type**	(1620) CAACGCCGCAAAGAGTTTGAAGCGAATTGGAAAGAACTTGACCCCAAGTAC
fd217	(1620) CAACGCCGCAAAGAGTTTAAAGCGAATTGAAAAGAACTTGACCCCAAGTAC
**Wild type**	(2948) CTGAGCCGATTCT____CT__C_G__TC______T_GAGGTGCTCCAACCA
fd213	(2948) CTGAGCCGATTCTACATCTACCTGAATCAGAGATTCGAGGTGCTCCAACCA
**Wild type**	(2948) CTGAGCCGATTCTCTCGTCTGAGGTGCTCCAACCAACTCGTCTGCCATCTG
fd214	(2948) CTGAGCCGAT_______TCTGAGGTGCTCCAACCAACTCGTCTGCCATCTG
**Wild type**	(2948) CTGAGCCGAT_TC__TCTCGT_CTGAGGTGCTCCAACCAACTCGTCTGCCA
fd215	(2948) CTGAGCCGATCTGAATCTCCCACCTACCAACTCCTACCAACTCGCTTGCCA

Comparison of wild-type sequence and corresponding mutated regions in CRISPR/Cas9 alleles of C04A11.4 (WY1208; *fd208*, *fd209*, and *fd210*), F56D12.6 (WY1209; *fd211* and *fd212*; WY1209), F48E8.5 (WY1211; *fd216* and *fd217*), and B0302.1 (WY1217; *fd213*, *fd214*, and *fd215*). Mutated regions are flanked upstream and downstream by wild-type nucleotides (black). Numbers indicate distance of the leftmost nucleotide shown from the start of the coding region (ATG) within the cDNA. Deleted regions are marked in red; insertions are in blue, and substitutions in yellow.
