# Supplementary material for: Use of a Sibling Subtraction Method for Identifying Causal Mutations in Caenorhabditis elegans by Whole-Genome Sequencing
Source: G3 (Bethesda). 2017 Dec 12;8(2):669–78. doi: 10.1534/g3.117.300135 (PMC5919755; doi:10.1534/g3.117.300135)
Supplement: Supplementary file 9 [file 669FileS1.pdf]

Oligonucleotides used in this study

For the following CRISPR targeting RNAs (crRNAs), target-specific sequences have a yellow background. For repair templates, altered nucleotides are in red, altered restriction endonucleases are underlined, and crRNA target sequences have a yellow background. For dsRNA template primers, target-specific sequences are underlined.

C04A11.4 (WY1208; *fd130*)
crRNA: 5'-CAGGAGUUCUGUUACGAAGGGUUUUAGAGCUAUGCU-3'
Alt-RTM CRISPR-Cas9 tracrRNA: IDT (CA#1072534) was used for all CRISPR injections.
Locus amplification: 5'-CCACCGAACAAACCCAATTG-3' and 5'-CGGAGCATTTAAGCCACCAT-3'
Locus sequencing: 5'-ATATTCGGTCATTGTGGCCC-3' and 5'-CTGGGTTTCACACTGCAACA-3'

F56D12.6 (WY1209; *fd131*)
crRNA: 5'-GCUCGAUCGGAAGCCGGCGCGUUUUAGAGCUAUGCU-3'
Locus amplification: 5'-GACTATCGCCTCAACTCAGA-3' and 5'-CTTCTCTATTTCCGCAGCTC-3'
Locus sequencing: 5'-GGAAGCGTGGATTCTGTGAT-3' and 5'-CAGCGAGCACCTTTTTACGA-3'
dsRNA primers: 5'-TAATACGACTCACTATAGGGAGAGGACGATTGGCGGAACAAAA-3' and
5'-TAATACGACTCACTATAGGGAGAGTGTCCAGGTTCGAATTTCC-3'

B0302.1 (WY1217; *fd139*)
crRNA: 5'-TGAGCCGATTCTCTCGTCTGGUUUUAGAGCUAUGCU-3'
Locus amplification: 5'-GCAACACGCCTATCACAGTT-3' and 5'-TCCGGAGAATCTGTTCTCTG-3'
Locus sequencing: 5'-TCAAATGTCGGACGAGGAGA-3' and 5'-CATTTAACTGAGGGTACCCG-3'
dsRNA primers: 5'-TAATACGACTCACTATAGGGAGACCATTCGAATATGCCCGCAA-3' and
5'-TAATACGACTCACTATAGGGAGATGAGTCACTGGTTTTGCCTG-3'

F48E8.5 (WY1211; *fd133*)
crRNA: 5'-GCAAAGAGTTTGAAGCGAATGUUUUAGAGCUAUGCU-3'
Locus amplification: 5'-GTTCTCAATCCGCGAGGCA-3' and 5'-TCGATTACAAGCCGAGTGCT-3'
Locus sequencing: 5'-GGAGCAGATTCTGAAGGAGA-3' and 5'-ACAGTTCTTGGCTTCCTCTG-3'
Repair template:
5'-CTTGTCGAGGATGACGTGCCGAATGTCAGATTCAACGCCGCAAAGAGTTTAAAGCGAA
TTGAAAAGAACTTGACCCCAAGGTGACAGGAAAATCTTTTTCACTATCCCAATTT-3'
(Creates DraI site)

W07E11.1
crRNA: 5'-TCAAACTTTGCACGGAGAACGUUUUAGAGCUAUGCU-3'
Locus amplification: 5'-CTCAGAAGCTGGAGTTGGAA-3' and 5'-TCTCTCCAGCAACTCCTCTA-3'
Locus sequencing: 5'-GTGCTCACGATGAACAACCT-3' and 5'-AATGGATCCGAACTCGAGCA-3'
Repair template:
GATGAGAAAATGTCACTTGAACACCTGCCCGGTTGGAGTCGCTACTCAAGATCTTGTTCTCCGTGCAAAGTTTGA
TGGAAAGCCAGAACACGTTGTCAACTATATG
(Creates BglII site)
dsRNA primers:

5'-TAATACGACTCACTATAGGGAGAACTTAAAGTGCGCTAACCCG-3' and
5'-TAATACGACTCACTATAGGGAGACCACCAGAGAGACATTTTCC-3'

T09B9.4
crRNA: 5'-GCTTGTTGGAATGTGCAATCGUUUUAGAGCUAUGCU-3'
Locus amplification: 5'-ATCAGGAGGAATTGATGCGC-3' and 5'-CGGCTTCGATACGAATAGCT-3'
Repair template:
ATCGTCAGCAACTCAACGAAACTGCCCATTTGCACATTGAAAACAAAAAGCTAGTTGGAATATGCAATCAAGAGA
GACGTCGATCGCAAGCTACAGAGGCTGAAGTCAAAAAATgttagtga
(Ablates HindIII site)
dsRNA primers:
5'-TAATACGACTCACTATAGGGAGAACACCGCTGCCTGATAATCA-3' and
5'-TAATACGACTCACTATAGGGAGATGACTTCAGCCTCTGTAGCT-3'

F53A9.7
crRNA: 5'-GCTTGTTGGAATGTGCAATCTCAGTGTGAACAACAGTTGG-3'
Locus amplification: 5'-GATCGGGCGTCAAAAATGGG-3' and 5'-ACTACACACCACTTTGAAGAAACA-3'
Repair template:
CATACGGAGGACACGGAGGATATGCTCCACCACCAGTCCATGGAGCTCCAGAATACATGCCACCGACGGTCGTT
CACACTGACGGAGGACATCACGGACACGTTGACACCCATCACCATGA
(Creates Hpy188III site)

T05A10.1
crRNA: 5'-GCTTGTTGGAATGTGCAATCATGCATGTCCGCTCCACCTG-3'
Locus amplification: 5'-ATAGCGATGGAACGACACCA-3' and 5'-GGATACCTGAGAGTTGGAAGG-3'
Repair template:
AACGATCACTATTGGCCTTCGCCGGATCGTCGATCATGCATGTCCGCTCCATCTGTAGCAATGCAACGTGACTTTG
ATGACCGAGGTGTGTGAAACTCGGATCTGGAT
(Creates SfcI site)

R04E5.10
crRNA: 5'-GCTTGTTGGAATGTGCAATCATTGAAATCTCCGAATTTAA-3'
Locus amplification: 5'-AGCACAAAGATGAATCAATCGTCA-3' and 5- TGACTGTACGATACGTGTGCAT-3'
Repair template:
ATATATTAAAAACAATTACAGATTCATTCATTGAAATCTCCGAATTTAATAGATGATATCGTTGCTACTCAAATCTA
CAGCTTCGATTTCTCCAAAAACACAAAGACAGTT
(Creates EcoRV site)

WGS, variant detection, and sibling subtraction method (SSM)

Our sequencing effort was designed to provide 20× average coverage of the *C. elegans* genome (105 Mb) to provide adequate depth to detect causative single-nucleotide polymorphisms (SNPs) in our experimental datasets. 2×125 paired-end library construction (150-bp insert) and sequencing were performed using the Tru-Seq DNA PCR-free Library Preparation Kit, and all

samples were sequenced in one lane on an Illumina HiSeq2000. We sequenced genomes from the non-mutant comparator for each mutant and also each of the five mutants described in this paper. All raw Illumina data are deposited in the NCBI Sequence Read Archive (http://trace. ncbi.nlm.nih.gov/Traces/sra) (BioProject ID PRJNA415825). Quality assessment was carried out with FastQC (*http://www.bioinformatics.babraham.ac.uk/projects/fastqc/*). Residual adapters and low-quality and short reads were removed via Trimmomatic v0.35 (Bolger *et al.* 2014).

Following quality control, we performed the remaining analyses with Galaxy (Afgan *et al.* 2016) following a modified version of the CloudMap protocol (Minevich *et al.* 2012). Protocols described below (*SSM Variant Detection* and *SSM Variant Subtraction*) can be accessed through the shared workflows on UseGalaxy.org. Individual tools used in Galaxy are noted in italics below (Li and Durbin 2009; Li *et al.* 2009; McKenna *et al.* 2010; Cingolani *et al.* 2012a; Cingolani *et al.* 2012b).

Tool	Function	Parameters
***Map with BWA for Illumina* [Galaxy version 1.2.3]**	Aligns reads to *the C. elegans* WS220 (ce10)] genome	• The parameters used for alignment were as follows: Library mate-paired = Paired-end, Maximum edit distance (aln -n) = 0<br>• Fraction of missing alignments given 2% uniform base error rate (aln -n) = 0.04<br>• Maximum number of gap opens (aln -o) = 1, Maximum number of gap extensions (aln -e) = -1<br>• Disallow long deletion within [value] bp towards the 3'-end (aln -d) = 16<br>• Disallow insertion/deletion within [value] bp towards the end (aln -i) = 5<br>• Number of first subsequences to take as seed (aln -l) = -1, Maximum edit distance in the seed (aln -k) = 2<br>• Mismatch penalty (aln -M) =3<br>• Gap open penalty (aln -O) = 11<br>• Gap extension penalty (aln -E) = 4<br>• Proceed with suboptimal alignments if there are no more than INT equally best hits (aln -R) = Null<br>• Disable iterative search (aln -N) = No<br>• Maximum number of alignments to output in the XA tag for reads paired properly (samse/sampe -n) = 3<br>• Maximum number of alignments to output in the XA tag for disconcordant read pairs (excluding singletons) (sampe -N) = 10, Maximum insert size for a read pair to be considered as being mapped properly (sampe -a) = 500<br>• Maximum occurrences of a read for pairing (sampe -o) = 100000<br>• Specify the read group for this file? (samse/sampe -r) = No<br>• Suppress the header in the output SAM file = No<br>• Job Resource Parameters = Use default job resource parameters

Filter SAM **[Galaxy version 1.0.0]**	Removes Orphaned and misaligned pairs	• 1: Flag<br>Read is paired = Yes<br>• 2: Flag<br>Read is mapped in proper pair = Yes
Add or Replace Groups **[Galaxy version 1.56.0]**	Alters BAM file read groups	• Read group ID (ID tag) = 1<br>• Read group sample name (SM tag) = rgSM,<br>• Read group library (LB tag) = rgLB<br>• Read group platform (PL tag) = rgPU<br>• Specify additional (optional) arguments = Use pre-set defaults<br>• Output bam instead of sam = Yes.
Realigner Target Creator **[Galaxy version 0.0.4]**	Identifies Intervals for local realignment	**[(1)]** Basic GATK options:<br>• How strict should we be in validating the pedigree information = STRICT<br>• Interval set rule = UNION<br>• Type of reads downsampling to employ at a given locus = NONE<br>• Type of BAQ calculation to apply in the engine = OFF,<br>• BAQ gap open penalty (Phred Scaled) = 40<br>• Use the original base quality scores from the OQ tag = No<br>• Value to be used for all base quality scores<br>• when some are missing = -1<br>• How strict should we be with validation = STRICT<br>Interval merging rule = ALL<br>• Disable experimental low-memory sharing functionality = No Makes the GATK behave non-deterministically that is, the random numbers generated will be different in every run = No.<br><br>**[(2)]** Basic Analysis options:<br>• Window size for calculating entropy or SNP clusters (windowSize) = 10<br>• Fraction of base qualities needing to mismatch for a position to have high entropy (mismatchFraction) = 0.15<br>• Minimum reads at a locus to enable using the entropy calculation (minReadsAtLocus) = 4<br>• Maximum interval size = 500
Indel Realigner **[Galaxy version 0.0.6]**	Performs Local alignment based on sequence intervals identified by *Realigner Target Creator*[Galaxy version 0.0.4]	Basic GATK and Basic Analysis options:<br>• LOD threshold above which the realigner will proceed to realign = 5.0<br>• Use only known indels provided as RODs = No<br><br>**[(1)]** Basic GATK options:<br>• How strict should we be in validating the pedigree information = STRICT, Interval set rule = UNION<br>• Type of reads downsampling to employ at a given locus = NONE<br>• Type of BAQ calculation to apply in the engine = OFF<br>• BAQ gap open penalty (Phred Scaled) = 40.0

		• Use the original base quality scores from the OQ tag = No<br>• Value to be used for all base quality scores, when some are missing = -1<br>• How strict should we be with validation = STRICT<br>• Interval merging rule = ALL<br>• Disable experimental low-memory sharing functionality = No Makes the GATK behave non-deterministically that is, the random numbers generated will be different in every run = No.<br><br>**[(2)]** Basic Analysis options:<br>• Percentage of mismatching base quality scores at a position to be considered having high entropy = 0.15<br>• Simplify BAM = No<br>• Consensus Determination Model = USE_READS<br>• Maximum insert size of read pairs that we attempt to realign = 3000<br>• Maximum positional move in basepairs that a read can be adjusted during realignment = 200<br>• Max alternate consensuses to try = 30<br>• Max reads (chosen randomly) used for finding the potential alternate consensus = 120<br>• Max reads allowed at an interval for realignment = 20000<br>• Don't output the original cigar or alignment start tags for each realigned read in the output bam = No
***Mark Duplicate Reads* [Galaxy version 1.56.0]**	Removes Duplicate reads	• Remove duplicates from output file = Yes<br>• Assume reads are already ordered = Yes<br>• Regular expression that can be used to parse read names in the incoming SAM file = [a-zA-Z0-9]+:[0-9]:([0-9]+):([0-9]+):([0-9]+).*<br>• The maximum offset between two duplicate clusters in order to consider them optical duplicates = 100
***Generate pileup from BAM dataset* [Galaxy version 1.1.2]**	Generates pileup file	• Whether or not to print the mapping quality as the last column = Do not print the mapping quality as the last column<br>• Whether or not to print only output pileup lines containing indels = print all lines<br>• Where to cap mapping quality = 30<br>• Call consensus according to MAQ model? = No
***Varscan* [Galaxy version 0.1]**	Allows for filtering variants across a range of allele frequency thresholds with SNPs and INDELs called separately	• Select your desired range of allele frequency thresholds = 100% - FA1, FA3; 90% -FA2, FA4 (Sup); 0% - FA1, FA3; 10% - FA2, FA4 (Non-Sup)<br>• Minimum read depth = 8 (Sup); 3 (Non-Sup)<br>• Minimum supporting reads =1<br>• Minimum base quality at a position to count a read =24<br>• Minimum frequency to call homozygote = FA1, FA3 - 100%; FA2, FA4 - 90%<br>• p-value threshold for calling variants = 0.99

***VCFCombine* [Galaxy Version 1.0.0_rc1.0]**	Combines SNP and INDEL VCF	• Select both SNP and INDEL files generated by the tool *Varscan*.
**VCF-VCF intersect [Galaxy version 1.0.0_rc1.0**	Performs Subtraction	• Mutant variant file was selected as the second VCF dataset and the NMSC variant file as the first VCF dataset. Then the following options were chosen to perform the subtraction:<br>• Union or intersection = Intersect<br>• Invert selection? = Yes<br>• compare records up to this many bp away (window size) = 30<br>• output whole loci when one alternate allele matches = Yes<br>• Advanced controls = Don't use advanced options.
***SnpEff* [Galaxy version 1.0]**	Annotates Variants	• Input format = VCF<br>• Output format = Tabular<br>• Genome = *Caenorhabditis elegans*: WS220.64<br>• Upstream/Downstream length = 10000 bases<br>• Filter homozygous/heterozygous changes = No Filter<br>• Filter homozygous/heterozygous changes = None<br>• Chromosomal position = Use default (based on input type).

Results from the above analysis can be downloaded from Galaxy and further subsetted according to individual need (*e.g.,* variants within coding regions or that occur on a particular chromosome). In addition, we recommend confirming candidate variants intended for experimental validation further by examining the individual non-mutant comparator and mutant alignments within a genome browser such as IGV (Robinson *et al.* 2011; Thorvaldsdóttir *et al.* 2013). This is to remove false positive candidates that are the result of a low number of reads in the Non-suppressed sibling strains. These are not called as variants in the Non-suppressed siblings and thus are not subtracted.

REFERENCES

Afgan, E., D. Baker, M. van den Beek, D. Blankenberg, D. Bouvier *et al.*, 2016 The Galaxy platform for accessible, reproducible and collaborative biomedical analyses: 2016 update. Nucleic Acids Res 44**:** W3-W10.

Bolger, A. M., M. Lohse and B. Usadel, 2014 Trimmomatic: a flexible trimmer for Illumina sequence data. Bioinformatics 30**:** 2114-2120.

Cingolani, P., V. M. Patel, M. Coon, T. Nguyen, S. J. Land *et al.*, 2012a Using Drosophila melanogaster as a Model for Genotoxic Chemical Mutational Studies with a New Program, SnpSift. Front Genet 3**:** 35.

Cingolani, P., A. Platts, L. Wang le, M. Coon, T. Nguyen *et al.*, 2012b A program for annotating and predicting the effects of single nucleotide polymorphisms, SnpEff: SNPs in the genome of Drosophila melanogaster strain w1118; iso-2; iso-3. Fly (Austin) 6**:** 80-92.

Li, H., and R. Durbin, 2009 Fast and accurate short read alignment with Burrows-Wheeler transform. Bioinformatics 25**:** 1754-1760.

Li, H., B. Handsaker, A. Wysoker, T. Fennell, J. Ruan *et al.*, 2009 The Sequence Alignment/Map format and SAMtools. Bioinformatics 25**:** 2078-2079.

McKenna, A., M. Hanna, E. Banks, A. Sivachenko, K. Cibulskis *et al.*, 2010 The Genome Analysis Toolkit: a MapReduce framework for analyzing next-generation DNA sequencing data. Genome Res 20**:** 1297-1303.

Minevich, G., D. S. Park, D. Blankenberg, R. J. Poole and O. Hobert, 2012 CloudMap: a cloud-based pipeline for analysis of mutant genome sequences. Genetics 192**:** 1249-1269.

Zuryn, S., S. Le Gras, K. Jamet and S. Jarriault, 2010 A strategy for direct mapping and identification of mutations by whole-genome sequencing. Genetics 186**:** 427-430.
