# Supplementary material for: Use of a Sibling Subtraction Method for Identifying Causal Mutations in Caenorhabditis elegans by Whole-Genome Sequencing
Source: G3 (Bethesda). 2017 Dec 12;8(2):669–78. doi: 10.1534/g3.117.300135 (PMC5919755; doi:10.1534/g3.117.300135)
Supplement: Supplementary file 11 [file 669SupplementalFigureLegend.pdf]

**Supplemental Figure Legends**

**Figure S1. Schematic diagram of F1 semi-clonal suppressor screen.** After mutagenesis of WY1145 [*nekl-2(fd81); nekl-3(gk894345); fdEx286* (pDF153*, nekl-3(+);* pTG96, SUR-5::GFP)], $P_0$ young-adult hermaphrodites were individually placed on small NGM plates and allowed to self-fertilize. Note that ~98.5% of $GFP^-$ WY1145 animals arrested with larval molting defects (Figure 1); for simplicity, rare adult escapers are not depicted. Several days later, $F_1$ hermaphrodites (two per plate) were transferred to new plates and allowed to reproduce. Plates with $F_2$ progeny were then scored for the presence of multiple $GFP^-$ adults, and candidate suppressed hermaphrodites were moved to new plates and allowed to reproduce.

**Figure S2. Schematic diagram of suppressor screen using counter-selection.** After mutagenesis of WY1255 [*nekl-2(fd81); nekl-3; fdEx303* (pDF153*, nekl-3(+);* pTG96, SUR-5::GFP, PMA122, $P_{hsp16.41}$*:peel-1*)], $P_0$ young-adult hermaphrodites were individually placed on large NGM plates and allowed to self-fertilize. Note that ~98.5% of $GFP^-$ WY1255 animals arrest with larval molting defects; for simplicity, rare adult escapers are not depicted. After ~7 days (approximately two generations), after the appearance of the $F_2$ larvae, plates were heat shocked to kill the large majority of $GFP^+$ hermaphrodites (gray worms). In some cases, heat shock was repeated ~4 days later to eliminate animals that had escaped death following the first heat shock. About 7 days after initial heat shock, plates were scored for the presence of large numbers of reproducing $GFP^-$ adults, which were then individually moved to additional plates.

**Figure S3. Crossing scheme for the isolation of strains for SSM/WGS.** Suppressed strains were crossed to starting strain WY1145 as described in the Results and Discussion, and *sup/sup* and *+/+* strains were isolated in the $F_2$ generation and used for WGS and SSM analysis. Also see Results and Discussion and Supplemental Methods sections for details.

**Figure S4. EMS density mapping of suppressed strains using CloudMap workflow.** EMS density mapping was carried out for each Suppressed DNA pool using the published CloudMap workflow, which uses Bayesian methods to call variants. The *x* axis indicates the location on each chromosome in mega bases (Mb). The number of EMS signature SNPs are indicated on the *y* axis; red bars indicate a 0.5-Mb region, and gray bars indicate a 1.0-Mb region. Black arrows indicate the locations of the identified causal mutations, and gray arrows indicate non-causal candidates identified by SSM.

**Figure S5. EMS density mapping of suppressed strains hybrid workflow.** EMS density mapping was carried out for each Suppressed DNA pool using our variant identification workflow, which determines variant calls based on allele frequencies, in conjunction with CloudMap tools. The *x* axis indicates the location on each chromosome in mega bases (Mb). The number of EMS signature SNPs are indicated on the *y* axis; red bars indicate a 0.5-Mb region, and gray bars indicate a 1.0-Mb region. Black arrows indicate the locations of the identified causal mutations, and gray arrows indicate non-causal candidates identified by SSM. The blue bars indicate genomic regions implicated by the mapping.

**Figure S6. EMS density mapping of WY1208 using a single comparator strain.** EMS density mapping was carried out for strain WY1208 as described in Figure S5, except that only one other suppressed strain (WY1211) was used to eliminate common variants prior to EMS density mapping. The *x* axis indicates the location on each chromosome in mega bases (Mb). The number of EMS signature SNPs are indicated on the *y* axis; red bars indicate a 0.5-Mb region, and gray bars indicate a 1.0-Mb region. Black arrows indicate the locations of the identified causal mutations, and gray arrows indicate non-causal candidates identified by SSM. The blue bar indicates the main genomic region implicated by the mapping, which contained 8 coding change variants.
